# Genome-Wide Analysis of the AP2/ERF Superfamily Genes and their Responses to Abiotic Stress in *Medicago truncatula*

**DOI:** 10.3389/fpls.2015.01247

**Published:** 2016-01-19

**Authors:** Yongjun Shu, Ying Liu, Jun Zhang, Lili Song, Changhong Guo

**Affiliations:** Key Laboratory of Molecular Cytogenetics and Genetic Breeding of Heilongjiang Province, College of Life Science and Technology, Harbin Normal UniversityHarbin, China

**Keywords:** *Medicago truncatula*, AP2/ERF transcription factors, abiotic stress, phylogenetic analysis, transcriptome analysis

## Abstract

The AP2/ERF superfamily is a large, plant-specific transcription factor family that is involved in many important processes, including plant growth, development, and stress responses. Using *Medicago truncatula* genome information, we identified and characterized 123 putative AP2/ERF genes, which were named as *MtERF1–123*. These genes were classified into four families based on phylogenetic analysis, which is consistent with the results of other plant species. MtERF genes are distributed throughout all chromosomes but are clustered on various chromosomes due to genomic tandem and segmental duplication. Using transcriptome, high-throughput sequencing data, and qRT-PCR analysis, we assessed the expression patterns of the MtERF genes in tissues during development and under abiotic stresses. In total, 87 MtERF genes were expressed in plant tissues, most of which were expressed in specific tissues during development or under specific abiotic stress treatments. These results support the notion that MtERF genes are involved in developmental regulation and environmental responses in *M. truncatula*. Furthermore, a cluster of DREB subfamily members on chromosome 6 was induced by both cold and freezing stress, representing a positive gene regulatory response under low temperature stress, which suggests that these genes might contribute to freezing tolerance to *M. truncatula*. In summary, our genome-wide characterization, evolutionary analysis, and expression pattern analysis of MtERF genes in *M. truncatula* provides valuable information for characterizing the molecular functions of these genes and utilizing them to improve stress tolerance in plants.

## Introduction

On a global scale, plant growth, and development are threatened by various abiotic stresses, such as extreme temperatures, drought, and high salinity. Due to environmental conditions continuously accumulating, plants are being confronted with increasingly serious challenges to their survival. Plants employ complex regulatory mechanisms to adapt to environmental stresses, undergoing physiological and biochemical changes in response to unfavorable conditions (Zhu, 2002; Chinnusamy et al., 2004; Katagiri, 2004). It is notable that plants contain numerous genes encoding transcription factors (TFs), which regulate the expression of downstream genes by binding to their *cis*-acting elements (Singh et al., 2002; Yamaguchi-Shinozaki and Shinozaki, 2006; Le Hir and Bellini, 2013). TFs play important roles in plant growth, development and responses to environmental stress by directly responding to stress or regulating the expression of downstream target genes, confirming their critical roles in plant life cycles. However, only a few TF families have been characterized outside of well-studied model plant systems, such as rice and Arabidopsis. The APETALA2/ethylene-responsive element binding factor (AP2/ERF) superfamily is one of the largest groups of TFs in plants. These TFs contain at least one AP2 domain. Based on the number of AP2 domains and other DNA binding domains, AP2/ERF TFs are classified into four families, including the AP2, ERF, RAV, and Soloist families (Cao et al., 2001; Sakuma et al., 2002; Mizoi et al., 2012). AP2 family members contain a double, tandemly repeated AP2 domain, while ERF family members contain a single AP2 domain. RAV family members have a single AP2 domain and an additional B3 domain, i.e., a DNA-binding domain commonly found in other TFs (Nakano et al., 2006; Mizoi et al., 2012; Li et al., 2015).

AP2/ERF TFs regulate a number of biological processes, such as plant growth, development, and responses to stress (Mizoi et al., 2012; Matías-Hernández et al., 2014). AP2 family members regulate the development of various plant tissues. For example, CRL5 is expressed in the stem region and regulates the crown root initiation process in rice (Kitomi et al., 2011). Some members of the AP2 family regulate reproductive development in Arabidopsis, including flower, ovule, and sepal development (Kunst et al., 1989; Krizek, 2009). AP2 TFs are also involved in fruit development and the ripening process in tomato and grapevine (Chung et al., 2010; Licausi et al., 2010; Sharma et al., 2010). The ERF family consists of two subfamilies (ERF and DREB), which participate in many developmental and stress response processes. ERF subfamily members, which bind to GCC-boxes, are involved in hormone signaling pathways, such as the ethylene, jasmonic acid, and salicylic acid pathways, which are important for plant development and stress responses (Fujimoto et al., 2000; Oñate-Sánchez and Singh, 2002; Andriankaja et al., 2007; Mantiri et al., 2008a). DREB subfamily members bind to dehydration-responsive element/C-repeat (DRE/CRT) elements, which are present in stress-responsive genes, including RD (responsive to desiccation, *RD29*) and COR (cold-regulated, *COR15*) genes (Cao et al., 2001; Xu et al., 2011). Therefore, many members of the DREB subfamily improve the stress tolerance of various plants under different environmental stress, including cold, drought, salinity stress, and so on.

Since the release of the whole-genome sequences of many plant species, the AP2/ERF superfamily has been successfully identified and investigated in plants including Arabidopsis, rice (Nakano et al., 2006), grapevine (Licausi et al., 2010), poplar (Zhuang et al., 2008), and soybean (Zhang et al., 2008). Genome-wide analysis of AP2/ERF TFs has helped elucidate their regulatory functions in plant growth, development and especially stress responses. *Medicago truncatula* is an annual, diploid legume plant. Due to its features, such as its small genome, self-pollination, high genetic transformation efficiency, nitrogen fixation, and so on, this plant has been chosen as a model legume for molecular genetic and genomic analyses. To date, only 37 AP2/ERF genes were identified based on EST sequences, which is far fewer than the number identified in any other plant species (Zhang et al., 2013b). The functions of AP2/ERF superfamily members in *M. truncatula*, especially those in responses to abiotic stress, have hardly been reported. ERN and ERN1, with an AP2 domain, were isolated from *M. truncatula*. Many studies have demonstrated that they have a regulation function in the process of nodulation, as do ERN2 and ERN3 (Middleton et al., 2007; Vernié et al., 2008; Hirsch et al., 2009). MtSERF1, regulated by the plant hormones auxin and cytokinin, plays an important role in somatic embryogenesis (Mantiri et al., 2008a,b). MtERF1-1, a member of the AP2/ERF B3 subgroup, has been shown to mediate resistance to root pathogens in *M. truncatula* (Anderson et al., 2010). WXP1 and WXP2 can improve drought tolerance in transgenic alfalfa by increasing leaf wax accumulation (Zhang et al., 2005, 2007). Pennycooke et al. (2008) isolated MtCBF1-3 from *M. truncatula*, and demonstrated their action against low temperature stress. MtCBF4 has been shown to respond to abiotic stresses, including cold, drought, salt, and ABA (Li et al., 2011). Interestingly, 12 potential MtCBF genes clustering on chromosome 6 have been shown to play a major role in tolerance to freezing by QTL mapping (Tayeh et al., 2013). However, their mechanism of doing so, has not yet been discovered. Their expression profiles during freezing remain to be determined. Recently, a draft of the *M. truncatula* genome sequence was completed and released (Young et al., 2011). A number of gene families have been analyzed based on this genome information, such as ARF and CCCH families, which were promoting Medicago Genus and legume genetic research (Zhang et al., 2013a; Gujaria-Verma et al., 2014; Shen et al., 2015).

In this study, we performed a comprehensive analysis of the AP2/ERF superfamily in *M. truncatula*, including phylogenetic analysis, chromosomal localization, gene duplication analysis, and expression profiling. We also characterized the functions of these TFs in the abiotic stress response via transcriptome analysis. The results of this study will be helpful for future investigations aimed at the functional characterization of these AP2/ERF TFs and their utilization for the genetic improvement of legumes.

## Materials and methods

### Identification and classification of the AP2/ERF genes in *Medicago truncatula*

*M. truncatula* genome and proteins sequences were downloaded from the JCVI website (*M. truncatula* Genome Project v4.0, http://www.jcvi.org/medicago/; Young et al., 2011), and Arabidopsis AP2/ERF gene sequences were downloaded from the DATF database (http://datf.cbi.pku.edu.cn; Guo et al., 2005). These Arabidopsis AP2/ERF sequences were utilized for BLAST (Altschul et al., 1990) searches against the *M. truncatula* genome sequence with the parameters of expected values ≤1E-3 and more than 80% coverage. All BLAST hits were retrieved and searched using the Hidden Markov Model (HMM) profile of the AP2 domain (PF002701), which was downloaded from the Pfam website (pfam.sanger.ac.uk; Finn et al., 2014). The AP2/ERF sequences were confirmed based on the presence of an AP2 domain, and all of the putative AP2/ERF proteins were aligned to Arabidopsis AP2/ERF proteins to classify them into different groups, as described by Nakano et al. (2006). Furthermore, all of the annotation information about putative AP2/ERF genes was retrieved from the *M. truncatula* genome website, and the number and distribution of introns in AP2/ERF genes were investigated using *M. truncatula* genome annotation information.

### Phylogenetic and conserved motif analysis of the AP2/ERF genes

Multiple alignments of candidate AP2/ERF protein sequences were carried out using ClustalW with default parameters (Thompson et al., 2002). Unrooted phylogenetic trees of all AP2/ERF proteins were generated with MEGA (V4.0) using the neighbor-joining (NJ) method with the following parameters: Poisson correction, pair-wise deletion and 1000 bootstrap replicates (Tamura et al., 2007). Conserved motifs in *M. truncatula* AP2/ERF TFs were identified using the motif finding tool MEME (Multiple EM for Motif Elicitation, V4.8.1; Bailey et al., 2006). MEME searching was performed across MtERF protein sequencing using the following parameters: (1) optimum motif width was set to ≥10 and ≤200; (2) the maximum number of motifs was set to identify 25 motifs; (3) occurrences of a single motif distributed among the sequences with model: zero or one per sequence (-mod zoops).

### Chromosomal localization and gene duplication analysis of the AP2/ERF genes

Positional information about all of the AP2/ERF genes was investigated, and diagrams of their chromosome locations in *M. truncatula* were drawn using the Circos software (http://circos.ca/; Krzywinski et al., 2009), revealing duplications between AP2/ERF genes in *M. truncatula*. If two genes with similarities of more than 85% were separated by four or fewer gene loci, they were identified as tandem duplications (TD). Others were identified as segmental duplications (SD), separated by more than five genes. In addition, duplications between the AP2/ERF genes were also identified and complemented using the PGDD database (http://chibba.agtec.uga.edu/duplication/; Lee et al., 2013). Duplicated genes between different chromosomes or loci were linked with colored lines in the diagrams using the Circos as previously described.

### *In silico* expression analysis of the AP2/ERF genes during plant development

Genome-wide transcriptome data from *M. truncatula* in different tissues during development were downloaded from the NCBI short read archive database (SRA database; http://www.ncbi.nlm.nih.gov, Accession numbers SRX099057–SRX099062). The transcriptome data were derived from six tissues, including roots, nodules, blades, buds, seedpods, and flowers. All transcriptome data were mapped to the *M. truncatula* genome using the TopHat (Trapnell et al., 2009), and the expression of MtERF genes was evaluated using the software Cufflinks as previously described (Trapnell et al., 2012). The expression data were analyzed and clustered using hierarchical cluster programs HCLUST of R and CLUSTERGRAM of Matlab (MathWorks, R2012a).

### Plant material and stress treatments

Seeds of *M. truncatula* (cv. Jemalong A17) were germinated and transferred to a mixture of perlite and sand (3:1, V/V). All seedlings were grown in a growth chamber (Conviron E15) at a temperature of 18 (night) and 24°C (day), a relative humidity of 60–80% and a 14/10 h photoperiod (daytime, 06:00–20:00). The seedlings were irrigated with half-strength Hoagland solution once every 2 days, and after 8 weeks, they were randomly divided into six groups for stress treatments. For cold stress (B group) and freezing stress (C group) treatment, the seedlings were transferred into another chamber with the temperature set at 4 or −8°C, respectively. For drought stress (D group) and salt stress (E group) treatment, the seedlings were treated with 300 mM mannitol or 200 mM NaCl solution, respectively. For ABA treatment (F group), the seedlings' leaves were sprayed with 100 μM ABA solution. Control (untreated, A group) and treated (B–F groups) seedlings were harvested at 3 h after treatment. For each group, five randomly chosen whole seedlings were pooled to form a biological replicate. All plant samples were frozen in liquid nitrogen and stored at −80°C until use.

### Transcriptome analysis of the response of the AP2/ERF genes to abiotic stress

Total RNA was extracted from six samples (one biological replicate sample per group) using the RNeasy Plant Mini Kit (Qiagen, Valencia, CA) following the manufacturer's instructions. The integrity of the RNA was assessed by formaldehyde agarose gel electrophoresis. Total RNA was quantified using a NanoDrop ND-1000 spectrophotometer (Thermo Fisher Scientific, Wilmington, DE, USA) and a Bioanalyzer 2100 (Agilent Technologies, CA). RNA Integrity Number (RIN) values were greater than 8.0 for all samples. Purified RNA samples were sent to BGI-Shenzhen Ltd. (Shenzhen, China) for construction of pair-end cDNA libraries and Illumina sequencing of abiotic stress-treated samples. Processing of raw data, removal of adapter sequences, base-calling, and quality value calculations were performed to produce clean data. Clean reads from six samples were mapped to the *M. truncatula* genome, and splice junctions were mapped using the TopHat. MtERF gene expression across six treatment samples (groups A–F) were evaluated using the Cufflinks software, and clustered using hierarchical cluster programs HCLUST of R and CLUSTERGRAM of Matlab (Mathworks, R2012a). Compared to controls, MtERF genes with fold changes ≥2 or ≤0.5 were identified as differentially expressed in response to abiotic stresses. The expression of MtERF genes was analyzed and visualized using the package ggplot2 of R platform.

### qRT-PCR validation of the AP2/ERF genes response to abiotic stress

Total RNA was isolated using the total RNA kit (Tiangen, Beijing, China) and then reverse transcribed into cDNA using the PrimeScript RT reagent Kit (Toyobo, Shanghai, China). qRT–PCR was performed using ABI 7300 Real-time Detection System (Applied Biosystems, USA) with SYBR Premix Ex TaqTM II (Toyobo, Shanghai, China). The PCR conditions were set as follows: 95°C for 2 min; 40 cycles of 95°C for 30 s and 55°C for 30 s; and 72°C for 1 min, and the experiments were repeated three biological replicates. The ΔΔC_T_ method was used to calculate relative expression levels of MtERF genes using GAPDH as reference gene. Primers of nine MtERF genes (randomly selected from DREB subfamily) and GAPDH gene used for qRT-PCR detection are listed in Table S1.

## Results

### Identification of the AP2/ERF TFs in *Medicago truncatula*

**Figure 1:**
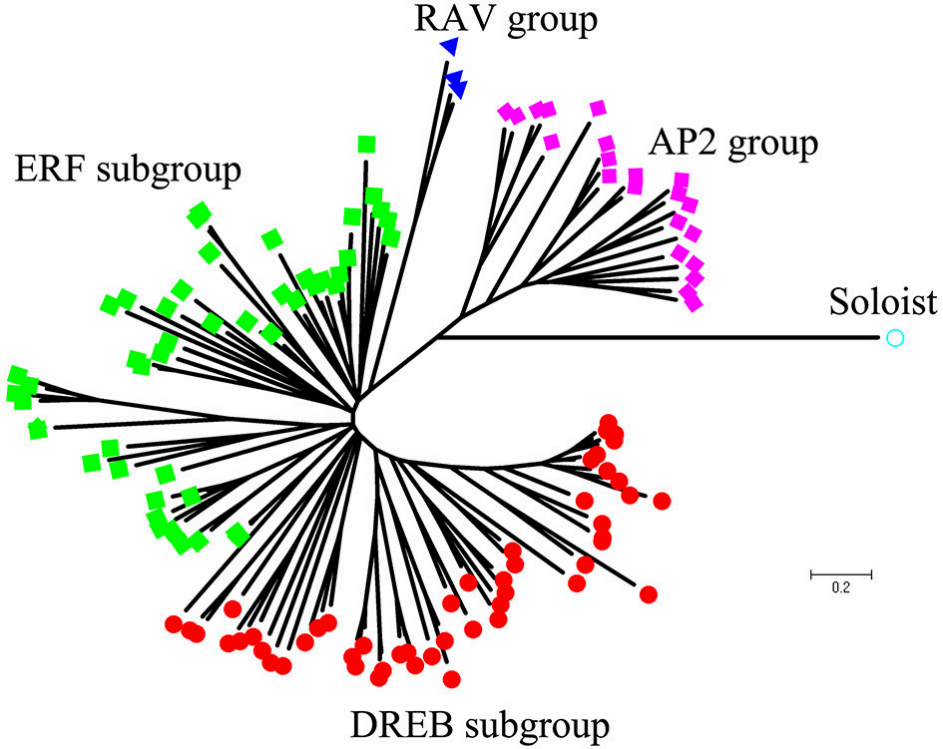
**Phylogenetic tree analysis of the AP2/ERF superfamily in ***Medicago truncatula*****.

**Figure 2:**
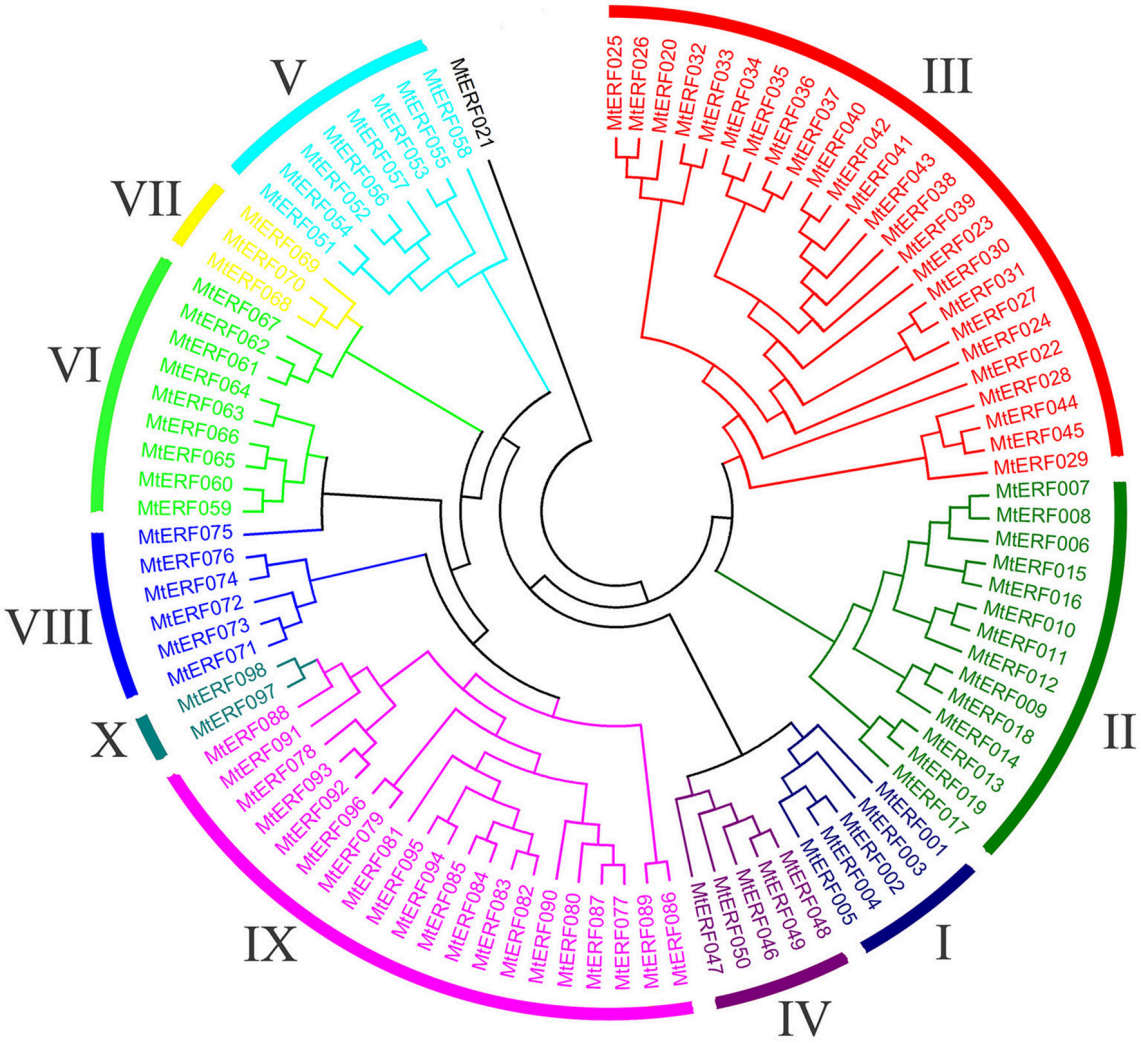
**Phylogenetic tree analysis of the ERF family in ***Medicago truncatula*****.

Using homology searches and domain confirmation, we identified 123 putative AP2/ERF TF genes in *M. truncatula*, designated as *MtERF001* to *MtERF123*, and we determined that these AP2/ERF genes encode putative proteins ranging from 120 to 689 aa in length (see Table 1). Among these TF genes, 98 genes with a single AP2/ERF domain were assigned to the ERF family, and based on the similarity of their encoded amino acid sequences, these genes were further classified into two subfamilies: 50 genes were identified as DREB subfamily members and 48 genes were identified as ERF subfamily members. Of the remaining MtERF genes, 21 genes were grouped into the AP2 family due to their tandemly repeated double AP2/ERF domain, and three genes were classified as RAV family members, as they encode proteins containing a single AP2/ERF domain together with a B3 domain. *MtERF123* is homologous to the Arabidopsis Soloist gene (At4g13040) and was therefore designated as Soloist, as shown in Figure 1. As Nakano et al. (2006) previously described, the genes of the ERF family can be subdivided into 10 groups according to their similarity to Arabidopsis ERF sequences, as shown in Figure 2. The DREB subfamily includes group I–IV, containing five, 14, 26, and five members, respectively, while the ERF subfamily consisted of group V–X, with eight, nine, three, six, 21, and two members, respectively. By BLAST search, nine AP2/ERF genes previously identified and totally characterized were also confirmed in the present study, see Table 2. The AP2/ERF family in *M. truncatula* has relatively few members compared with other plants, such as Arabidopsis (147), soybean (148), grapevine (149), rice (180), and poplar (202) (Nakano et al., 2006; Zhang et al., 2008; Zhuang et al., 2008; Licausi et al., 2010). The numbers of AP2 and RAV family members varies little among species, ranging from 18 to 29 and from three to six, respectively, which contributes little to the reduction in the total number identified in *M. truncatula*. This small number may arise from the reduced number of ERF family members, as there are 98 members of the ERF family in *M. truncatula* and 122, 122, 169, and 145 members in Arabidopsis, grapevine, soybean, rice, and poplar, respectively.

**Table 1:** **List of all MtERF genes identified in the ***Medicago truncatula*** genome**.

**Gene name**	**Gene locus**	**Chromosome location**	**AA**	**Introns**	**Family group**
MtERF001	Medtr1g090170	chr1:40395351-40396983	403	0	I
MtERF002	Medtr3g074130	chr3:33468981-33470875	340	0	I
MtERF003	Medtr5g009410	chr5:2245117-2246644	320	0	I
MtERF004	Medtr5g062700	chr5:25984370-25986874	371	0	I
MtERF005	Medtr8g090350	chr8:38012580-38014120	286	0	I
MtERF006	Medtr1g014780	chr1:3551204-3551899	231	0	II
MtERF007	Medtr1g014800	chr1:3562399-3563058	219	0	II
MtERF008	Medtr1g014860	chr1:3588962-3589621	219	0	II
MtERF009	Medtr1g019110	chr1:5681145-5682526	180	0	II
MtERF010	Medtr2g043020	chr2:18729389-18730846	203	0	II
MtERF011	Medtr2g043030	chr2:18732619-18733579	202	0	II
MtERF012	Medtr2g043050	chr2:18742621-18743617	214	0	II
MtERF013	Medtr3g072610	chr3:32669719-32670657	220	0	II
MtERF014	Medtr3g102100	chr3:47037850-47039084	172	0	II
MtERF015	Medtr3g105480	chr3:48645234-48645926	230	0	II
MtERF016	Medtr3g105510	chr3:48653623-48654357	244	0	II
MtERF017	Medtr5g008790	chr5:1925879-1926821	162	0	II
MtERF018	Medtr5g016750	chr5:6026308-6027413	182	0	II
MtERF019	Medtr5g058470	chr5:24173719-24174782	298	0	II
MtERF020	Medtr1g006660	chr1:70778-71741	257	0	III
MtERF021	Medtr1g060910	chr1:26538338-26539007	173	0	III
MtERF022	Medtr1g101550	chr1:45868926-45870250	232	0	III
MtERF023	Medtr1g101600	chr1:45888187-45888799	202	0	III
MtERF024	Medtr2g085015	chr2:36077334-36078017	227	0	III
MtERF025	Medtr2g101340	chr2:43561394-43562060	206	0	III
MtERF026	Medtr3g110205	chr3:51237707-51238569	187	0	III
MtERF027	Medtr4g102660	chr4:42553246-42554433	220	0	III
MtERF028	Medtr5g008550	chr5:1814617-1815198	193	0	III
MtERF029	Medtr5g008590	chr5:1836845-1837408	187	0	III
MtERF030	Medtr5g010910	chr5:3021016-3021690	224	0	III
MtERF031	Medtr5g010940	chr5:3037835-3038624	229	0	III
MtERF032	Medtr6g088405	chr6:33589051-33589710	219	0	III
MtERF033	Medtr6g088425	chr6:33601826-33602912	235	0	III
MtERF034	Medtr6g465420	chr6:23246544-23247851	267	1	III
MtERF035	Medtr6g465430	chr6:23250898-23252309	248	1	III
MtERF036	Medtr6g465450	chr6:23257085-23258239	245	1	III
MtERF037	Medtr6g465460	chr6:23265303-23267001	271	1	III
MtERF038	Medtr6g465510	chr6:23283223-23283906	208	0	III
MtERF039	Medtr6g465530	chr6:23289566-23290162	198	0	III
MtERF040	Medtr6g465690	chr6:23390649-23391838	260	1	III
MtERF041	Medtr6g465990	chr6:23582824-23583464	193	1	III
MtERF042	Medtr6g466000	chr6:23586674-23587485	227	0	III
MtERF043	Medtr6g466130	chr6:23633759-23634451	230	0	III
MtERF044	Medtr7g117690	chr7:48856582-48857258	171	0	III
MtERF045	Medtr8g027465	chr8:9776124-9776786	177	0	III
MtERF046	Medtr3g112440	chr3:52690417-52691100	227	0	IV
MtERF047	Medtr5g082950	chr5:35786315-35787316	333	0	IV
MtERF048	Medtr5g083330	chr5:35974606-35975715	299	2	IV
MtERF049	Medtr5g083340	chr5:35977177-35979527	407	1	IV
MtERF050	Medtr7g092190	chr7:36509882-36510748	288	0	IV
MtERF051	Medtr1g012470	chr1:2480152-2480838	197	1	V
MtERF052	Medtr2g103700	chr2:44648214-44649280	175	1	V
MtERF053	Medtr3g106290	chr3:49102873-49104079	189	1	V
MtERF054	Medtr3g107380	chr3:49532324-49533111	208	1	V
MtERF055	Medtr4g008860	chr4:1725344-1727163	195	1	V
MtERF056	Medtr4g010640	chr4:2428183-2429442	213	1	V
MtERF057	Medtr4g114570	chr4:47100854-47101969	214	1	V
MtERF058	Medtr7g085810	chr7:33273705-33274484	259	0	V
MtERF059	Medtr1g105400	chr1:47424522-47425616	364	0	VI
MtERF060	Medtr1g110970	chr1:50101581-50102669	344	1	VI
MtERF061	Medtr3g090760	chr3:41196627-41198512	355	0	VI
MtERF062	Medtr5g009620	chr5:2360490-2362483	309	0	VI
MtERF063	Medtr5g057647	chr5:23782045-23782944	299	0	VI
MtERF064	Medtr5g057810	chr5:23868998-23869897	299	0	VI
MtERF065	Medtr8g023680	chr8:8584553-8585984	365	0	VI
MtERF066	Medtr8g023700	chr8:8600406-8602622	363	1	VI
MtERF067	Medtr8g099215	chr8:41747534-41748589	313	0	VI
MtERF068	Medtr2g105380	chr2:45435775-45438505	367	2	VII
MtERF069	Medtr2g435590	chr2:13770126-13772657	259	1	VII
MtERF070	Medtr8g022820	chr8:8110327-8113473	382	2	VII
MtERF071	Medtr2g014300	chr2:4034815-4036032	172	0	VIII
MtERF072	Medtr3g053690	chr3:21358224-21361945	225	2	VIII
MtERF073	Medtr4g078710	chr4:30411899-30413146	218	0	VIII
MtERF074	Medtr5g032820	chr5:14158435-14159523	362	0	VIII
MtERF075	Medtr5g085130	chr5:36737809-36738348	179	0	VIII
MtERF076	Medtr7g084370	chr7:32557164-32558339	282	0	VIII
MtERF077	Medtr1g043350	chr1:16251768-16252550	218	0	IX
MtERF078	Medtr1g048610	chr1:18708193-18709593	231	0	IX
MtERF079	Medtr1g069960	chr1:30641535-30642361	149	0	IX
MtERF080	Medtr1g070000	chr1:30671540-30672306	225	0	IX
MtERF081	Medtr1g070070	chr1:30735948-30736700	146	0	IX
MtERF082	Medtr1g074230	chr1:32986163-32986625	138	0	IX
MtERF083	Medtr1g074250	chr1:32992934-32993329	131	0	IX
MtERF084	Medtr1g074280	chr1:33002942-33003304	120	0	IX
MtERF085	Medtr1g074290	chr1:33011049-33012641	161	1	IX
MtERF086	Medtr1g074310	chr1:33017698-33018264	144	0	IX
MtERF087	Medtr1g074370	chr1:33042222-33043578	213	0	IX
MtERF088	Medtr2g015050	chr2:4419705-4420718	276	0	IX
MtERF089	Medtr2g438180	chr2:15403524-15403925	133	0	IX
MtERF090	Medtr4g054360	chr4:19719627-19720433	268	0	IX
MtERF091	Medtr4g100380	chr4:41378394-41379900	268	0	IX
MtERF092	Medtr4g100420	chr4:41398779-41400388	307	0	IX
MtERF093	Medtr4g100450	chr4:41405946-41407325	307	0	IX
MtERF094	Medtr7g096700	chr7:38809591-38810090	143	0	IX
MtERF095	Medtr7g096750	chr7:38822540-38823486	138	0	IX
MtERF096	Medtr7g096810	chr7:38839796-38840343	128	0	IX
MtERF097	Medtr7g020980	chr7:6566578-6569664	176	1	X
MtERF098	Medtr7g021010	chr7:6580663-6583753	176	1	X
MtERF099	Medtr1g017400	chr1:4844540-4848969	660	7	AP2
MtERF100	Medtr1g049140	chr1:18978653-18984068	343	6	AP2
MtERF101	Medtr2g093060	chr2:39643101-39647675	468	9	AP2
MtERF102	Medtr2g098180	chr2:41962851-41966348	525	8	AP2
MtERF103	Medtr2g460730	chr2:25049516-25052612	363	6	AP2
MtERF104	Medtr3g103460	chr3:47751102-47755318	658	7	AP2
MtERF105	Medtr4g007770	chr4:1228729-1234955	324	6	AP2
MtERF106	Medtr4g061200	chr4:22613977-22618175	469	9	AP2
MtERF107	Medtr4g065370	chr4:24560917-24564307	546	9	AP2
MtERF108	Medtr4g094868	chr4:39153135-39156804	522	9	AP2
MtERF109	Medtr4g097520	chr4:40188319-40192060	655	7	AP2
MtERF110	Medtr4g127930	chr4:53232820-53237003	512	8	AP2
MtERF111	Medtr4g130270	chr4:54266443-54269143	359	7	AP2
MtERF112	Medtr5g015070	chr5:5176273-5179958	544	8	AP2
MtERF113	Medtr5g016810	chr5:6063009-6066677	517	9	AP2
MtERF114	Medtr5g031880	chr5:13680655-13684967	514	7	AP2
MtERF115	Medtr7g080460	chr7:30617123-30621534	689	8	AP2
MtERF116	Medtr7g091390	chr7:36109176-36113347	414	7	AP2
MtERF117	Medtr8g020510	chr8:7209112-7212535	574	7	AP2
MtERF118	Medtr8g044040	chr8:16855707-16858718	387	5	AP2
MtERF119	Medtr8g044070	chr8:16864525-16870371	417	5	AP2
MtERF120	Medtr1g093600	chr1:41951396-41953202	384	0	RAV
MtERF121	Medtr1g116920	chr1:52806975-52807897	298	0	RAV
MtERF122	Medtr5g053920	chr5:22189479-22191258	378	0	RAV
MtERF123	Medtr8g012655	chr8:3721647-3726436	243	6	Soloist

**Table 2:** **MtERF genes and their functions as identified and characterized in previous reports**.

**Gene ID**	**Gene Name**	**Accession Number**	**Function**	**References**
MtERF002	WXP2	TC94548	Drought stress	Zhang et al., 2007
MtERF004	WXP1	TC107019	Drought and freezing stress	Zhang et al., 2005
MtERF023	MtCBF4	HQ110079	Abiotic stress (ABA, drought, salt, and cold)	Li et al., 2011
MtERF031	MtCBF1	EU139868	Low temperature stress	Pennycooke et al., 2008
MtERF040	MtCBF2	EU139867	Low temperature stress	
MtERF042	MtCBF3	EU139866	Low temperature stress	
MtERF058	ERN	TC99463	Nodulation process	Middleton et al., 2007
	ERN1	EU038802	Nodule development	Vernié' et al., 2008; Hirsch et al., 2009
MtERF091	MtERF1-1	TC144328	Resistance to root pathogens	Anderson et al., 2010

### Phylogenetic and conserved motif analysis of the AP2/ERF TFs in *Medicago truncatula*

To determine the evolutionary relationships between AP2/ERF family proteins in *M. truncatula*, a phylogenetic tree was constructed based on alignment of full-length sequences of MtERF proteins. The phylogenetic tree confirms that MtERF TFs could be classified into four groups, shown as Figure 1, which is consistent with the classification results obtained using homology searches as described above. The phylogenetic relationships of the 98 ERF family members were assessed in depth. The results show that these genes (except *MtERF21*) could be divided into 10 groups, as described by Nakano et al. (2006). As shown in Figure 2, groups I–IV were identified as DREB subfamily members, while groups V–X were characterized as ERF subfamily members.

**Figure 3:**
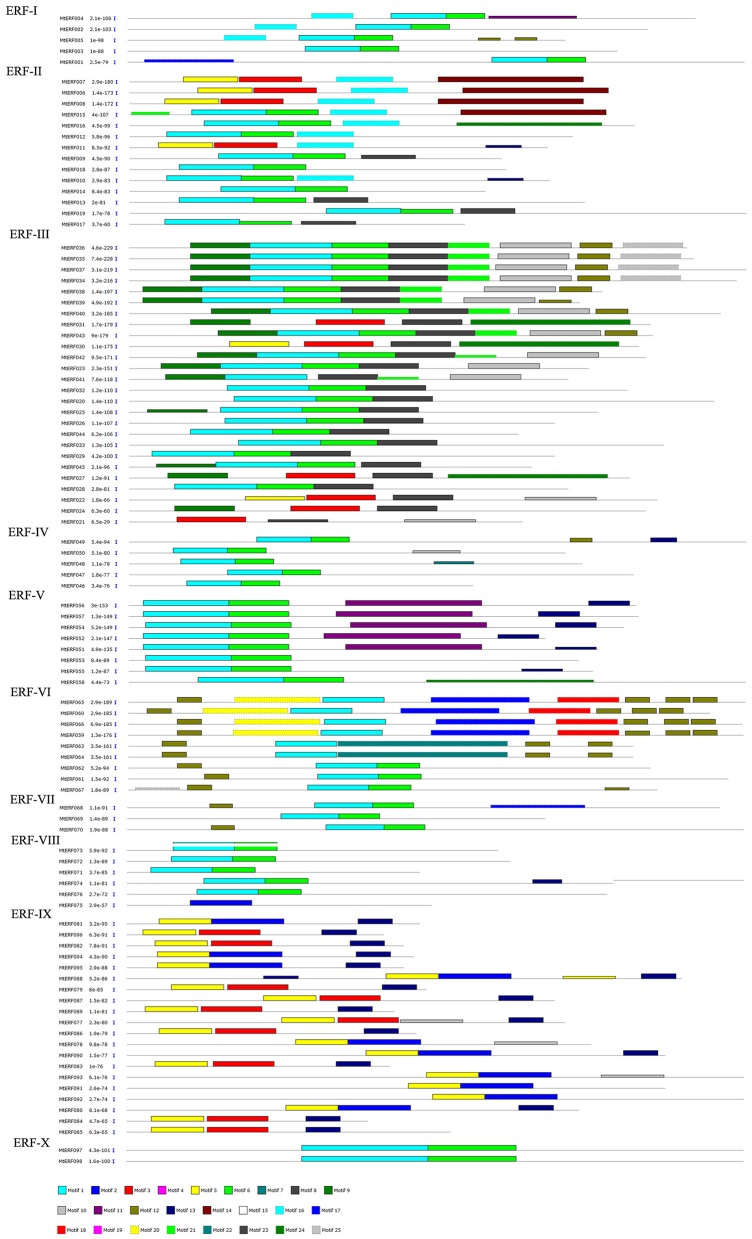
**Distribution of conserved motifs within ERF family in ***Medicago truncatula*****.

**Figure 4:**
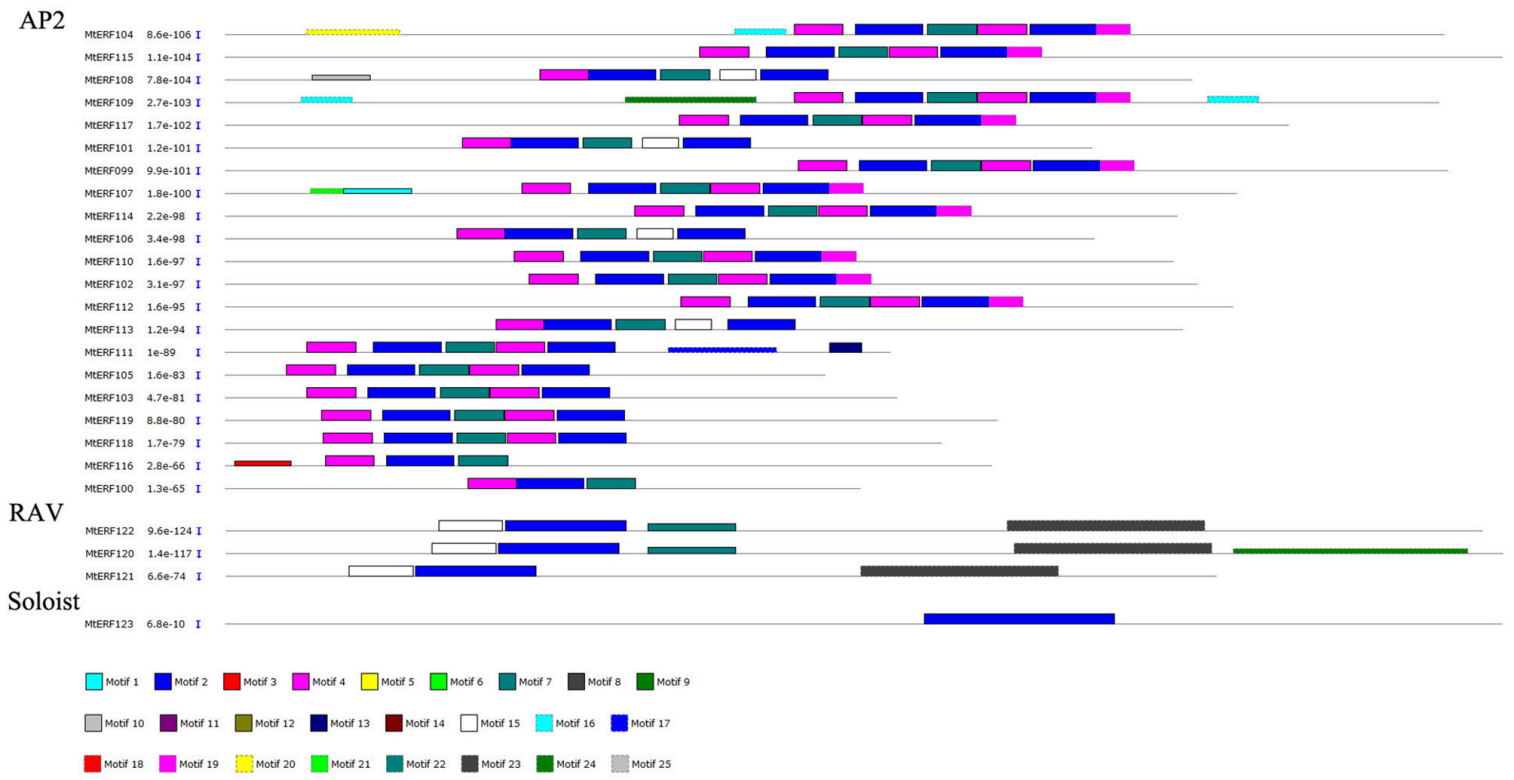
**Distribution of conserved motifs within AP2 and RAV families in ***Medicago truncatula*****.

The conserved motifs in AP2/ERF family proteins in *M. truncatula* were investigated using MEME, revealing a total of 25 conserved motifs (designated motifs 1–25), as shown in Figures 3, 4. Motifs 1–6 were found to be similar to the AP2/ERF domain region, while the remaining motifs corresponded to regions outside of the AP2/ERF domain region, which are distributed in specific clades in the phylogenetic tree. Proteins in the same group or subgroup contain similar motifs, while the motifs are divergent among different groups or subgroups. For example, motif 23 is only present in each member of the RAV family, which indicates that this motif is specific to the RAV family. Similarly, motif 19 is shared by members of the AP2 family, motif 16 is present in groups ERF-I and II, and the ERF II group contains motif 14 while the ERF I group does not. Motifs 8–10 are specific to the ERF III group, and motif 8 is shared by each member of the ERF III group. Finally, while motif 9 and 10 are shared by most members (16/26, 62%), motif 11 and 13 are conserved in the ERF V group and motif 12 is shared by groups ERF VI and VII. These results indicate that most motifs are distributed among specific groups, which is correlated with their functional divergence (see Figures S1–S4).

### Chromosomal locations and duplication of the AP2/ERF TFs in *Medicago truncatula*

**Figure 5:**
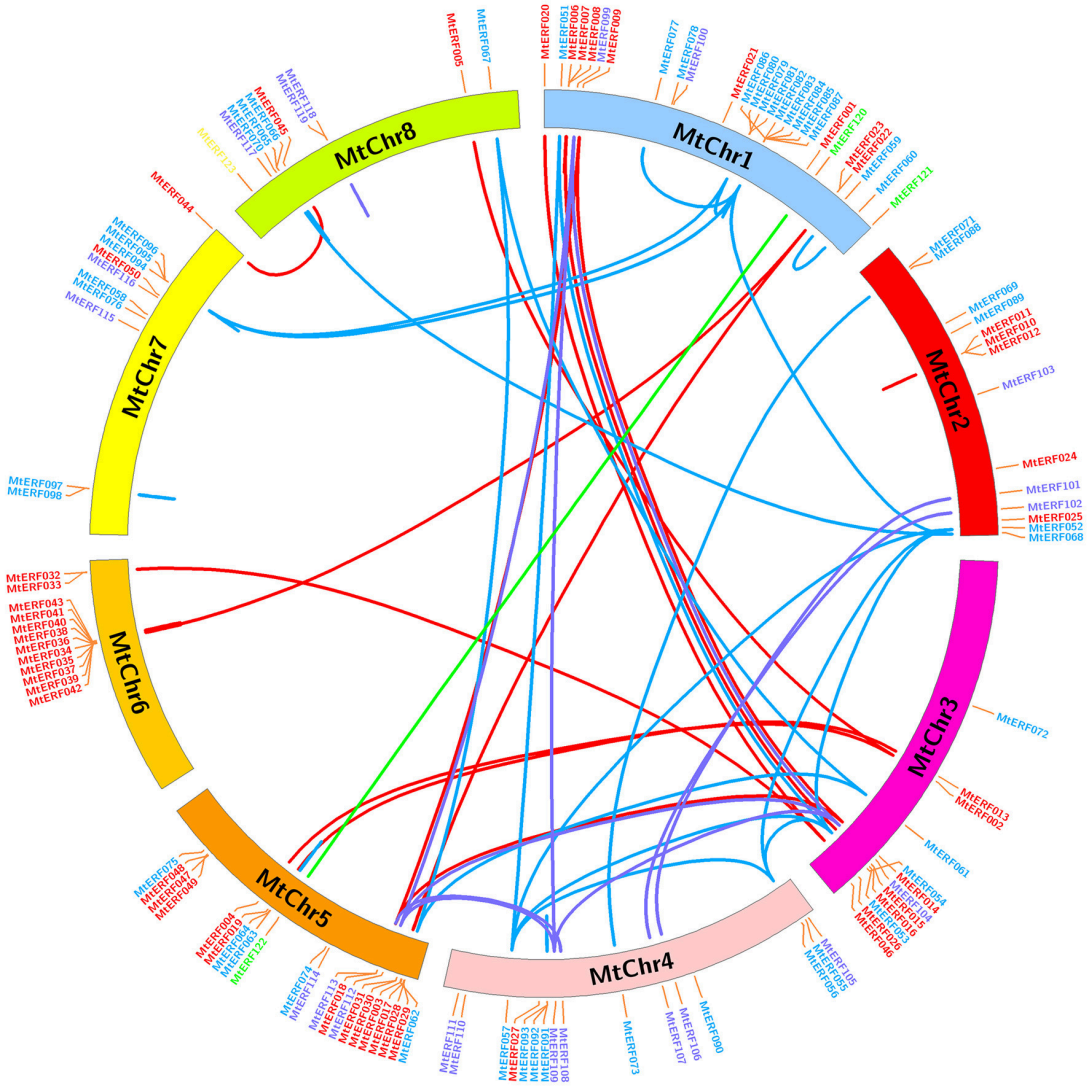
**Chromosomal distribution and expansion analysis of MtERF genes in ***Medicago truncatula*****. Red lines show duplications between members of the DREB subfamily, blue lines show duplications between members of the ERF subfamily, purple lines show duplications between members of the AP2 family and green lines show duplications between members of the RAV subfamily.

The 123 AP2/ERF TF genes are distributed throughout the eight chromosomes of *M. truncatula*; their physical locations on chromosomes are shown in Figure 5. Each *M. truncatula* chromosome contains some AP2/ERF genes in numbers ranging from 10 to 27. Chromosomes 1 and 5 have the highest number of AP2/ERF genes (27 and 21 genes, respectively), while chromosomes 7 and 8 contain the fewest genes (11 and 10, respectively). The AP2/ERF genes are not randomly distributed on each chromosome, as there are some gene clusters “hot regions” on the chromosomes. For example, chromosome 6 contains 10 AP2/ERF genes (MtERF34–43) in a short chromosome region (~390 kb), and chromosomes 1, 2, and 5 contain similar gene clusters, as shown in Figure 5. In addition, using gene duplication analysis, we identified 38 pairs of gene duplications, which arose from tandem duplications and segmental duplications. Tandem duplications produced MtERF gene clusters or hot regions, such as the MtERF34–43 cluster on chromosome 6 and the MtERF10–12 cluster on chromosome 2. Segmental duplication produced many homologous AP2/ERF genes on different chromosomes, which expanded the numbers of MtERF genes from different groups. For example, MtERF52, 54, 56, and 57 from group ERF-V are distributed on different chromosomes (MtERF52 on chromosome 2, MtERF54 on chromosome 3, MtERF56, and 57 on chromosome 4), which are products of genome segmental duplication.

### Expression profiles of MtERF genes in *M. truncatula* tissues

**Figure 6:**
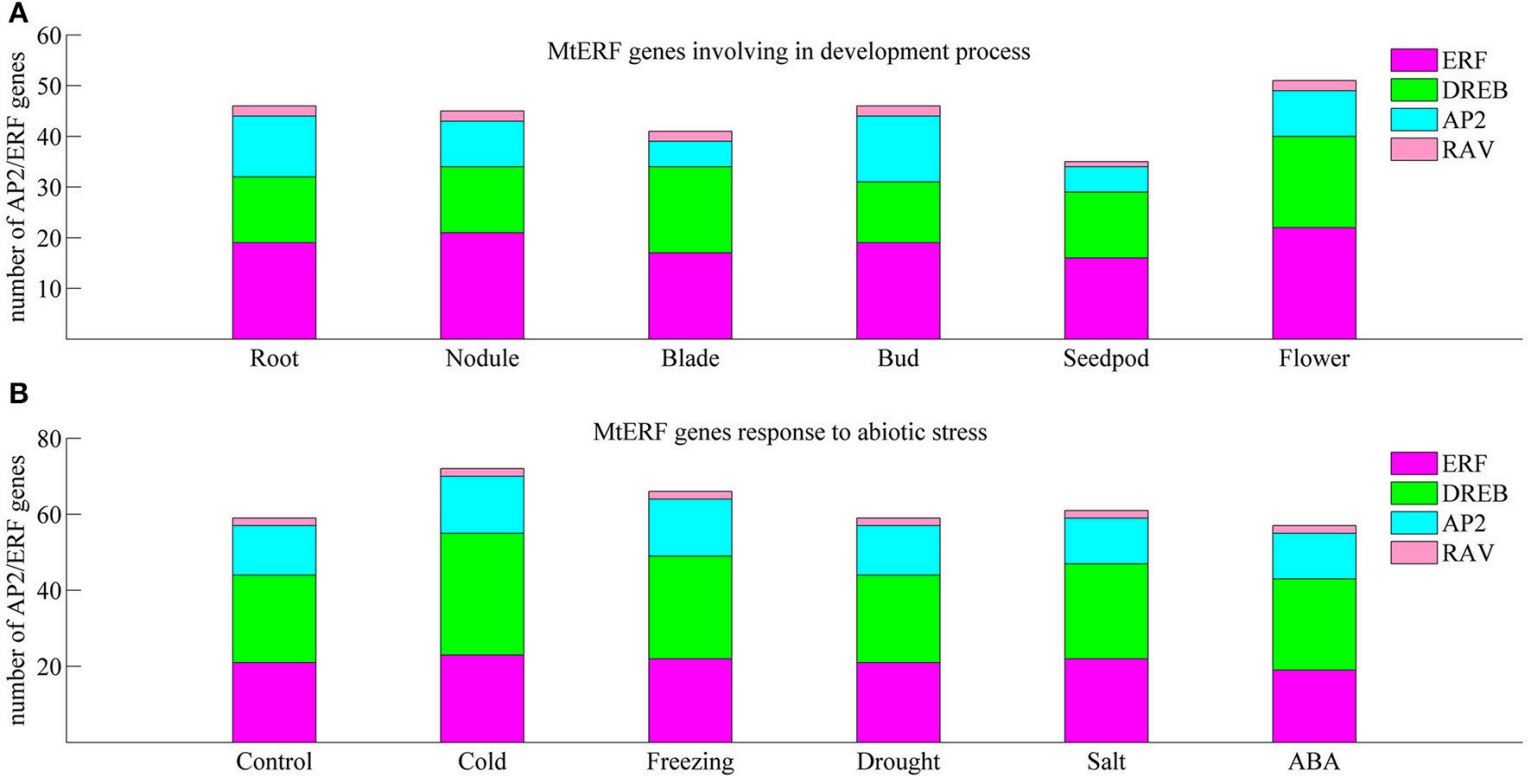
**Number of differentially expressed MtERF genes involved in tissue development (A) and stress responses (B)**.

**Figure 7:**
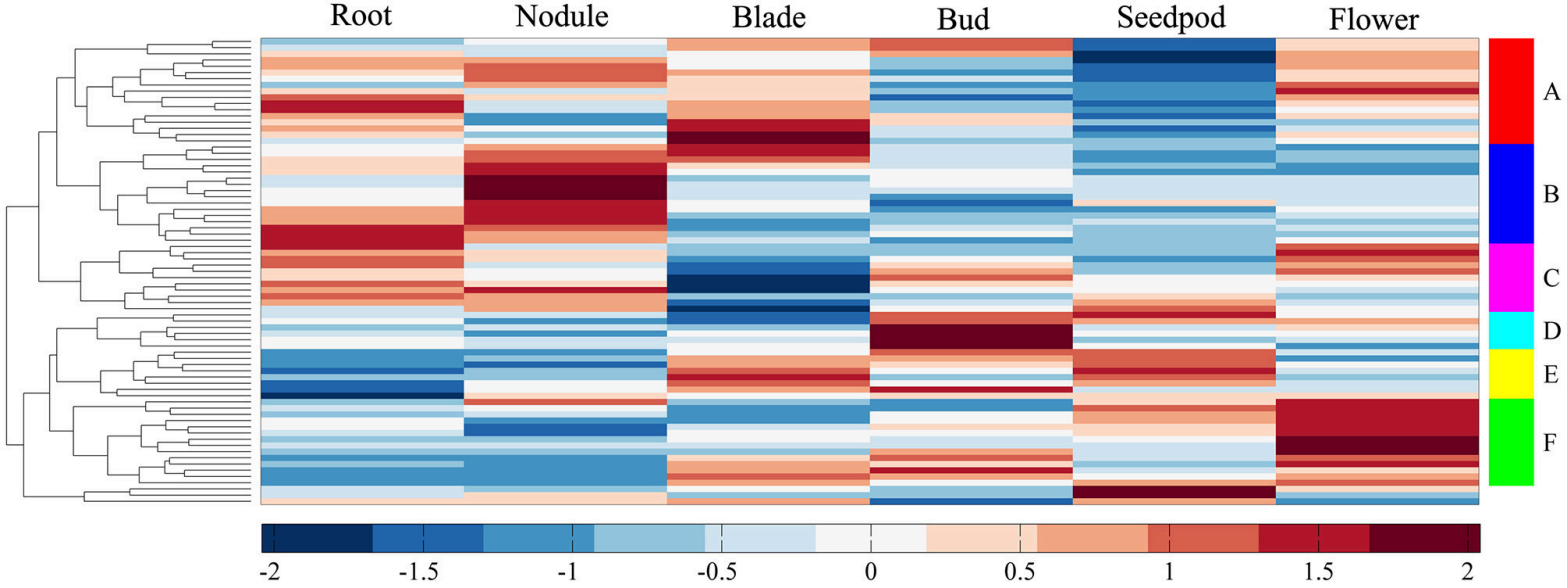
**Expression profile cluster analysis of MtERF genes involved in tissue development**.

We investigated the expression profiles of MtERF genes in various tissues using high-throughput sequencing data from NCBI, including root, nodule, blade, bud, seedpod, and flower tissues, revealing that 75 MtERF genes were expressed in at least one of six tissues. Of these, the expression of 46 genes was detected in root tissue, 46 genes in nodule tissue, 42 genes in blade tissue, 47 genes in bud tissue, 36 genes in seedpod tissue, and 52 genes in flower tissue (see Figure 6A). To further elucidate the transcription patterns of MtERF genes, their expression patterns were clustered across six tissues, as shown in Figure 7. Among these MtERF genes, group A (17 MtERF genes) were highly expressed across all six tissues, while the others exhibited tissue-specific profiles. For example, group B (16 MtERF genes) were highly expressed in root and nodule tissues and not in other tissues. Similarly, group D (six MtERF genes) were specifically expressed in buds, group E (eight MtERF genes) in seedpod and blades and group E (14 MtERF genes) in flowers.

**Figure 8:**
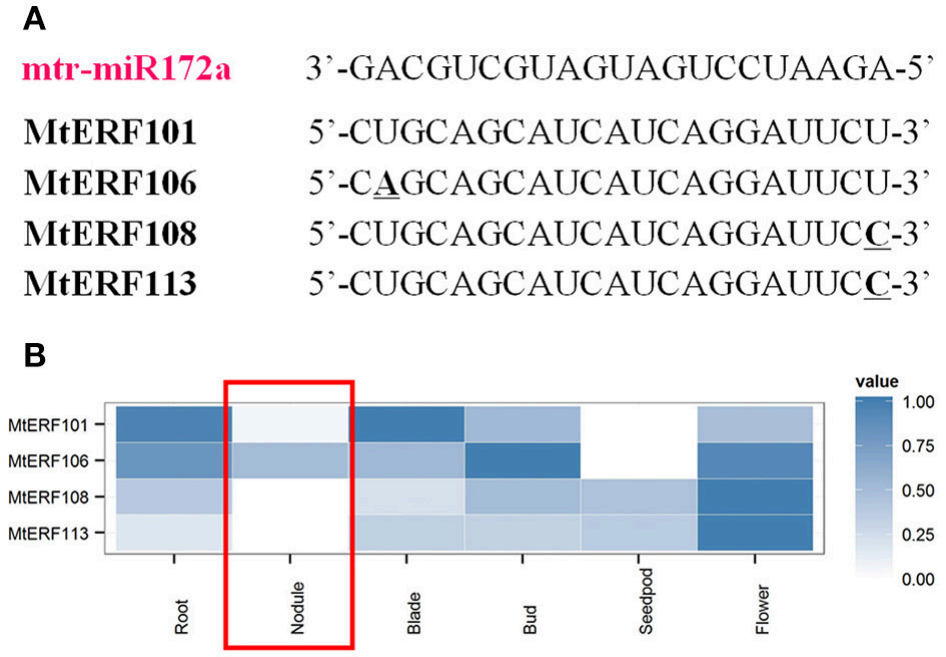
**Putative miR172 target sites in mRNAs of AP2 family genes**. **(A)** miRNA172 cleavage sites in MtERF genes; **(B)** expressional profiles of MtERF genes in tissue development.

Recently, researchers have demonstrated that microRNA miR172 plays an important role by targeting AP2 TFs in the root nodule symbiosis of legumes (Reynoso et al., 2013; Wang et al., 2014; Nova-Franco et al., 2015). To determine potential regulatory mechanisms of miRNAs and AP2 TFs in the nodulation process of *M. truncatula*, we submitted *M. truncatula* miRNA genes (download from miRBase, Van Peer et al., 2014) and MtERF genes to the psRNATarget website (Dai and Zhao, 2011) for the identification of potential target sites. There were four MtERF genes (MtERF101, 106, 108, and 113) of the miR172 family with unambiguously identified cleavage sites (see Figure 8A, and Table S2). Based MtERF genes expression from high-through data, we found that four target MtERF genes had low expression in nodules. Indeed, two of them (MtERF108 and MtERF113) were not expressed at all (Figure 8B).

### Expression responses of MtERF genes to abiotic stress

**Figure 9:**
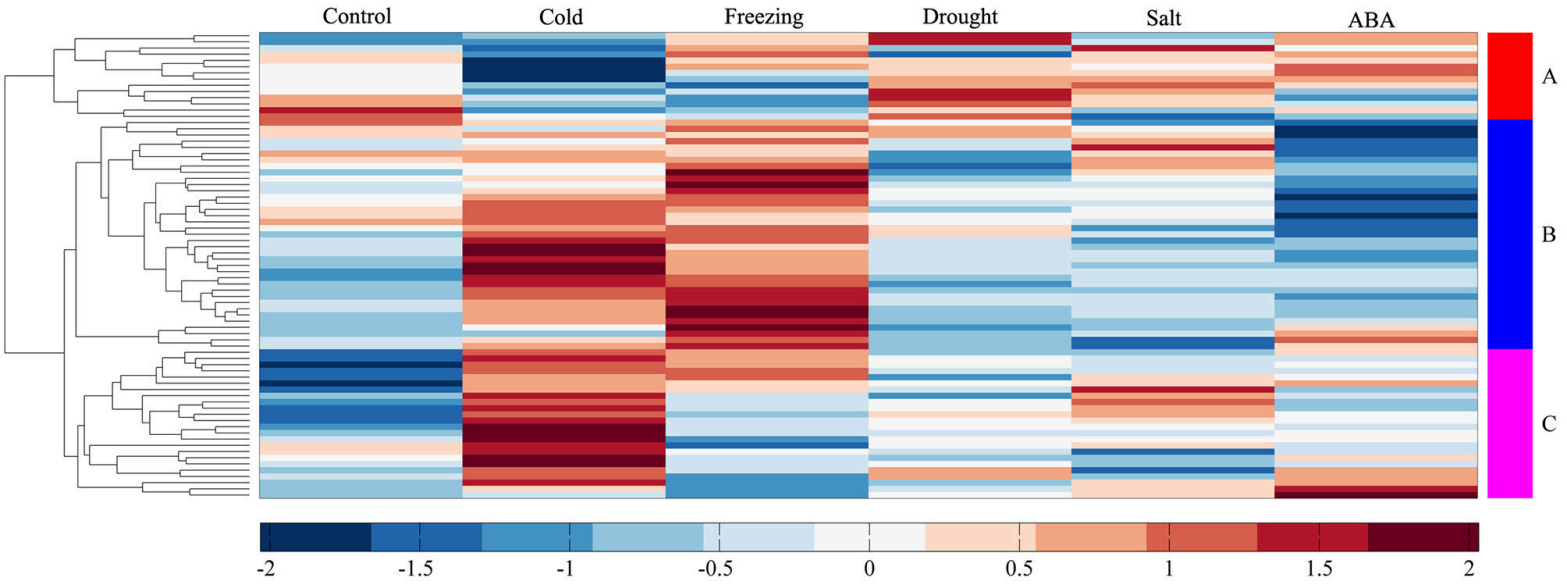
**Expression profile cluster analysis of MtERF genes responses to abiotic stresses**.

To investigate the molecular functions of MtERF genes in response to abiotic stress, we performed RNA-seq to detect the expression levels of AP2/ERF TF genes under different stresses, including cold, freezing, drought, salt and ABA. In total, more than 135 M clean reads from control (23,357,742), cold (20,437,484), freezing (23,469,784), drought (22,672,350), salt (21,993,278), and ABA (23,441,046) treated libraries were obtained, respectively; all clean data were submitted to the NCBI SRA database (Accession numbers: SRX1056987–92). After mapping these reads to the *M. truncatula* genome, we determined that the expression of 75 MtERF genes was detected in at least one library. As shown in Figure 6B, 60 MtERF genes were expressed in the control library, 73 MtERFs under cold stress, 67 MtERFs under freezing stress, 60 MtERFs under drought stress, 62 MtERFs under salt stress, and 58 MtERFs under ABA treatment. Based on expressional profiles of MtERF genes responses to abiotic stresses, they were clustered into three groups, as shown in Figure 9. Among these MtERF genes, group A (14 MtERF genes) were highly expressed by most abiotic stresses, except cold stress. While group B (37 MtERF genes) were highly expressed in response to cold and/or freezing stress, but not in response to other stresses. Group C (24 MtERF genes) were specifically expressed in response to cold stress, fewer in freezing stress and others. Compared to the control library, we identified 48 MtERF genes that were differentially expressed under at least one stress condition, as shown in Figure S5. A total of 35 MtERF genes were differentially expressed under cold stress, while 29 MtERF genes were differentially expressed under freezing stress. A total of 12, 12, and 23 MtERF genes were differentially expressed under drought stress, salt stress and ABA stress, respectively, which was consistent with the MtERF genes clustering results. Notably, four MtERF genes (*MtERF022, MtERF023, MtERF043*, and *MtERF073*) were up-regulated under all stress conditions, suggesting that they play important roles in the response of *M. truncatula* to abiotic stress.

**Figure 10:**
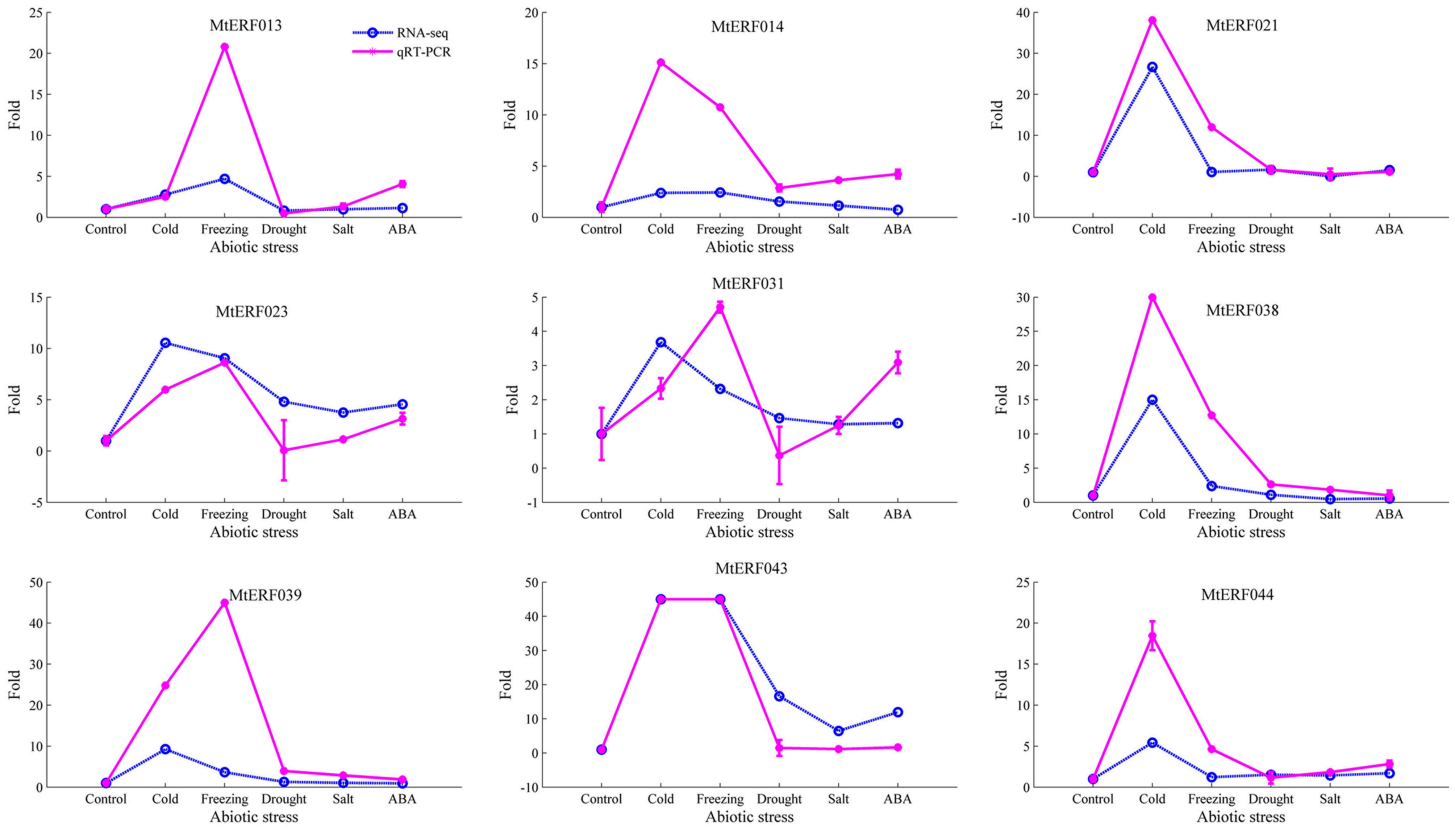
**qRT-PCR validation of MtERF genes in the response to abiotic stress**. Fold changes more than 45 were set as 45 for good plot (including two genes, MtERF039, and 043).

To further confirm the RNA-seq results of these MtERF genes to abiotic stresses, qRT-PCR was performed for nine MtERF genes from the DREB subfamily under abiotic stresses. The expression patterns of most of the MtERF genes in the qRT-PCR analysis were consistent with RNA-Seq analysis, but the magnitude of the fold changes varied between RNA-seq and qRT-PCR experiments (Figure 10). The means of the correlation coefficients of the qRT-PCR validations and the RNA-seq results for the MtERF genes were as high as 0.83, implying that our RNA-seq results were highly reliable.

## Discussion

In this study, we performed a comprehensive search for AP2/ERF TF genes throughout the Medicago genome; 123 MtERF genes were identified and characterized. Previously, Zhang et al. (2013b) identified 37 AP2/ERF genes using EST sequences, whereas we characterized 123 AP2/ERF members from the ERF, AP2, and RAV families. Compared to other plants, the number of AP2/ERF genes in Medicago is slightly lower than that in Arabidopsis (148), soybean (147), cucumber (131), and grapevine (149) and much lower than that in rice (180) and poplar (202). This is most likely due to the lower number of DREB and ERF subfamily members, as there are 98 members in Medicago and 122 in Arabidopsis, 120 in soybean, 122 in grapevine, 145 in rice, and 169 in poplar. By contrast, the number of AP2 and RAV family members is highly conserved among higher plants. For example, 21 members of the AP2 family were identified in this study, while 18, 26, 20, 29, and 26 AP2 genes were identified in Arabidopsis, soybean, grapevine, rice, and poplar, respectively.

In general, TFs always harbor some important conserved domains and motifs for their regulatory function. In MtERFs, 25 motifs were identified based on the MEME results; six motifs (motifs1–6) related to the AP2 domain. Among these motifs, motif1 harbored the largest region of the AP2 domain, including the whole of the β-sheet region and part of the α-helix region (G[VI]R, Gx_4_E, WLG, and AYD elements, as described by Nakano et al., 2006). The other five motifs were short AP2 domains. Motif4 and motif5 contained G[VI]R and Gx_4_E elements, while motif2, motif3, and motif6 contained WLD and AYD elements. All MtERFs contained at least one of the six motifs, indicating that the AP2 domain is highly conserved in MtERF genes. Besides the AP2 domain-related motifs, the other 19 motifs outside the AP2 domain were present in group-specific distributions. For example, motif17 was specifically present in the ERF VI group (also called the ERF B5 group), and contained (L/F) DLN (L/F) xP residues. It was identified as an ERF-associated amphiphilic repression (EAR) motif, essential for repression function (Ohta et al., 2001). Meanwhile, a SP[TV]SVL motif was characterized. It had the potential to be phosphorylated by mitogen activated protein kinase (MAPK) (Nakano et al., 2006). Motif17 may have specific repression functions by virtue of the phosphorylation of the ERF V group. Motif13 contained a unique “EDLL” motif, and was present in the ERF V and IX groups. The “EDLL” motif was previously characterized as a transcriptional activation domain in AP2/ERF TF (Tiwari et al., 2012), implying its participation in the activating function of the ERF V and IX groups (previously called the B6 and B3 groups, respectively). Motif8 was highly conserved in all members of the ERF III group and in four members of the ERF II group (in total, including 30 out of the 45 members of the DREB subfamily). It contained two conserved residue features: LPRP and D[IV]QAA. They have been identified as being essential regulation signatures of the response to various stresses in plants (Albrecht et al., 2001; Qu and Zhu, 2006). Their expression response to abiotic stresses has been shown by RNA-seq analysis. However, except for the three motifs 8, 13 and 17, and the six AP2 domain related motifs (motifs 1–6), the function of the other 16 conserved motifs (65% of the total) identified in present study is uncertain. Their roles need to be further characterized.

AP2/ERF genes in various plant species are differentially expressed in different tissues, indicating that they play important roles in plant tissue development. Genes in the ERF subfamily play many diverse roles, such as functioning in the response to hormonal stimuli and regulating developmental processes in various angiosperms. Meanwhile, members of the AP2 family participate in the regulation of developmental processes, such as flower development and meristem determinacy (Krizek, 2009). In the current study, 75 MtERF genes were found to be expressed in at least one tissue based on high-throughput sequencing data analysis, including DREB (26/50, 52%), ERF (31/48, 64.58%), AP2 (15/21, 71.43%) and RAV (2/3, 66.67%), genes and one Soloist gene (1/1, 100%). Except for two families with fewer members (RAV and Soloist), most members of the ERF and AP2 families participate in the regulation of tissue determinacy. When we compared the expression of MtERF genes in these two families, we found that ERF genes had higher expression levels than AP2 genes, which may be the result of the higher intron content of the AP2 family. Each member of the AP2 family has five to nine introns, while most ERF genes lack introns (74/98) and others contain only one or two introns. Due to the small number of introns, genes in the ERF family respond more quickly and are expressed at higher levels than AP2 family genes. Of the six tissues examined in Medicago, MtERF genes were most highly expressed in flower tissue, which is consistent with previous reports (Krizek, 2009; Matías-Hernández et al., 2014).

Like other leguminous plants, *M. truncatula* has established a symbiotic relationship with nitrogen fixing rhizobial bacteria, resulting in the formation of specialized lateral organs, called nodules. Nodulation is a complex developmental process involving many molecular signals constituting a genetic regulatory network. TFs, including GRAS, AP2/ERF, NF-Y, and others, have been shown to have important roles in controlling the expression of early nodulation genes (ENODs) and in regulating the later steps of the rhizobial symbiotic interaction (Combier et al., 2006; Andriankaja et al., 2007; Middleton et al., 2007; Vernié et al., 2008; Hirsch et al., 2009; Zanetti et al., 2010; Soyano et al., 2013; Laloum et al., 2014; Baudin et al., 2015). The ERF V group, previously called the B6 group in other studies, contains eight members, four of which (MtERF 52, 53, 55, and 58) were highly expressed in nodule tissue (Figure S6). MtERF 55 and 58 were only expressed in nodule tissue. This strongly suggests that these genes play unique and important roles in regulating root development and the symbiotic associations with rhizobia. We found that MtERF58 was homologous to ERN and ERN1 (Table 2). Previous researchers have demonstrated that the ERN gene (similar to ERN1), which is homologous to RAP2.11, interacts with GRAS factors in the regulation of NF-elicited gene transcription rhizobial infection (Andriankaja et al., 2007; Middleton et al., 2007; Vernié et al., 2008). MtERF55 is homologous to *PtaERF003*, which has been identified as promoting root development in *Populus* (Trupiano et al., 2013). These results confirm the potential of the ERF V group for the regulation of nodulation. Their functions in the development of other tissues need further investigation.

Nodulation is a complex process in *M. truncatula*, involving the ERF V group and other AP2/ERF genes, with different regulation patterns. As previously mentioned, many researchers have found that miRNA miR172 is also involved in the nodule development of legumes (Wang et al., 2014; Nova-Franco et al., 2015). It regulates the nodulation process by targeting AP2/ERF TFs, and repressing their expressions. In so doing, it is a negative regulator of root nodulation. In the present study, four AP2/ERF genes (Figure 8A), belonging to the AP2 family, were identified as potential targets of miR172 with unambiguous target sites. Based on expression analysis *in silico*, we found that these MtERF genes had low levels of expression in nodules, implying that they were potentially down-regulated by miR172 in the nodulation process. These results show the divergent functions of MtERF genes in nodulation. MtERF58, with high levels of expression, is a positive regulator of nodulation in *M. truncatula*; as confirmed in many studies. However, members of the AP2 family (MtERF 101, 106, 108, 113) were cleaved by miR172, and have low levels of expression in nodules. They may be important repressors of nodulation. This is well-characterized in soybeans and in common beans (Wang et al., 2014; Nova-Franco et al., 2015). However, its function in *M. truncatula* needs further confirmation.

When adapting to various environmental conditions, plants employ many TF families involved in regulating a wide range of defense responses to environmental clues. AP2/ERF superfamily TFs with ERF domains can bind to GCC-box elements or DRE motifs, thereby regulating gene expression in response to biotic or abiotic stress (Fujimoto et al., 2000; Cao et al., 2001). As previously shown, MtCBF1-3 isolated from *M. truncatula*, has one AP2 domain, and have key functions in cold acclimatization in *M. truncatula* and the related species, *M. falcate* (Pennycooke et al., 2008; Table 2). Similarly, Li et al. (2011) identified the role of MtCBF4 in responding to abiotic stresses, including cold, drought, and salt. Studies in which it was over-expressed conferred improved drought and salt tolerance. Notably, Tayeh et al. (2013) identified a major role for QTL (Mt-FTQTL6) in tolerance against freezing, demonstrated 40% of the phenotypic variation by QTL mapping, and characterized 12 potential MtCBF genes clustering in the Mt-FTQTL6 region. However, the expression profiles of these MtCBF genes in response to freezing stress are still poorly understood, and their function in freezing tolerance in *M. truncatula* is unknown. In this study, we analyzed the expression of MtERF genes under different stress conditions via transcriptome sequencing. We identified 75 MtERF genes that were expressed under various stress treatments, among which 63 were also expressed during tissue development, while 12 were specifically expressed under stress treatment, as shown in Figure S7. Compared to the control samples, 48 MtERF genes were found to be differentially expressed in response to abiotic stress (see Figure S5), and most MtERF genes were induced by abiotic stress. These MtERF genes include DREB (23/48, 47.92%), ERF (15/48, 31.25%), AP2 (7/48, 14.58%), RAV (2/48, 4.17%), and one Soloist (1/48, 2.08%) gene, the DREB and ERF subfamilies are the largest groups that were responsive to abiotic stress. We found that the DREB and ERF subfamilies were more sensitive to abiotic stress, while ERF and AP2 members were active in tissue development. Among these MtERF genes, 43 MtERF genes were responsive to cold or freezing stress (see Figure S8), while 30 MtERF genes were responsive to salt, drought or ABA-induced stress, as shown in Figure S9. A total of 25 MtERF genes commonly respond to abiotic stress. These include MtERF22, 23, 43, amongst others. MtERF23, which was called MtCBF4 in the report by Li et al. (2011), was previously identified as being induced by cold, drought, salt, and ABA stresses. Our RNA-seq data and qRT-PCR experiments confirmed its high expression under abiotic stresses, (see Figure 10). Of the MtERF genes, 21 were specifically induced or repressed by low temperature stresses, including the previously reported MtCBF 1-3 (Pennycooke et al., 2008). In our study, these were called MtERF 31, 40, and 42, respectively. Most of them are from the DREB subfamily (see Figure S5). Their expression profiles indicate that MtERF genes play more important roles in regulating the response to abiotic stresses *M. truncatula*, especially DREB subfamily critical function to low temperature stress. Interestingly, 10 MtERF genes (MtERF34–43) are clustered on chromosome 6, and they were tandemly duplicated within an ~393 kb region, which was previously identified by Tayeh et al. (2013). In the present study, we identified and characterized 10 MtERF genes, belonging to the DREB subfamily. Two previously identified potential MtCBF genes were excluded because the aligning region with Arabidopsis ERF genes was less than 80%. Moreover, our RNA-seq profiles demonstrated that six of these MtERF genes (also called MtCBF genes) were highly up-regulated under both cold and freezing stress (see Figure S5), and qRT-PCR had confirmed their high expression in cold and freezing stresses (MtERF38, 39, and 43, see Figure 10). The expression profiles suggested important and complementary potential roles of the MtERF gene cluster on chromosome 6 under abiotic stresses, especially its major contribution to freezing tolerance in *M. truncatula*.

## Conclusions

In summary, we identified 123 MtERF genes from the *M. truncatula* genome sequence. We investigated the classification, evolution, and tissue-specific expression of these MtERF genes, revealing that MtERF genes broadly participate in the regulation of plant tissue development. Meanwhile, we identified 48 candidate MtERF genes that may be involved in abiotic stress responses, their expression profiles were confirmed by qRT-PCR experiment. In particular, a tandem array of MtERF genes on chromosome 6 was identified and found to function in the response to cold and freezing stress, as revealed by RNA-seq analysis. The results of this study will be useful for identifying and characterizing these genes. Further functional analyses of these genes will be performed in the future to enable them to be used for transgenic applications.

### Conflict of interest statement

The authors declare that the research was conducted in the absence of any commercial or financial relationships that could be construed as a potential conflict of interest.

## Supplementary material

Figure S1 **Conserved motifs identified from members of the DREB subfamily in ***Medicago truncatula*****.

Figure S2 **Conserved motifs identified from members of the ERF subfamily in ***Medicago truncatula*****.

Figure S3 **Comparison of amino acid sequences of the AP2/ERF domains in the DREB subfamily**.

Figure S4 **Comparison of amino acid sequences of the AP2/ERF domains in the ERF subfamily**.

Figure S5 **Differential expression analysis of MtERF genes involved in the response to abiotic stress**.

Figure S6 **Expression profile cluster analysis members of MtERF V group in tissue development**.

Figure S7 **Venn diagram of shared expression MtERF genes between tissues development and stresses response**.

Figure S8 **Venn diagram of shared expression MtERF genes between cold stress and freezing stress**.

Figure S9 **Venn diagram of shared expression MtERF genes among salt, drought and ABA stresses**.

Table S1 **List of qRT-PCR validation primers used in the present study**.

Table S2 **Summary of miR172 targeting AP2/ERF TF genes in ***Medicago truncatula*****.
