# Supplementary figures and images for: Genome-Wide Analysis of the AP2/ERF Superfamily Genes and their Responses to Abiotic Stress in Medicago truncatula
Source: Front Plant Sci. 2016 Jan 19;6:1247. doi: 10.3389/fpls.2015.01247 (PMC4717309; doi:10.3389/fpls.2015.01247)

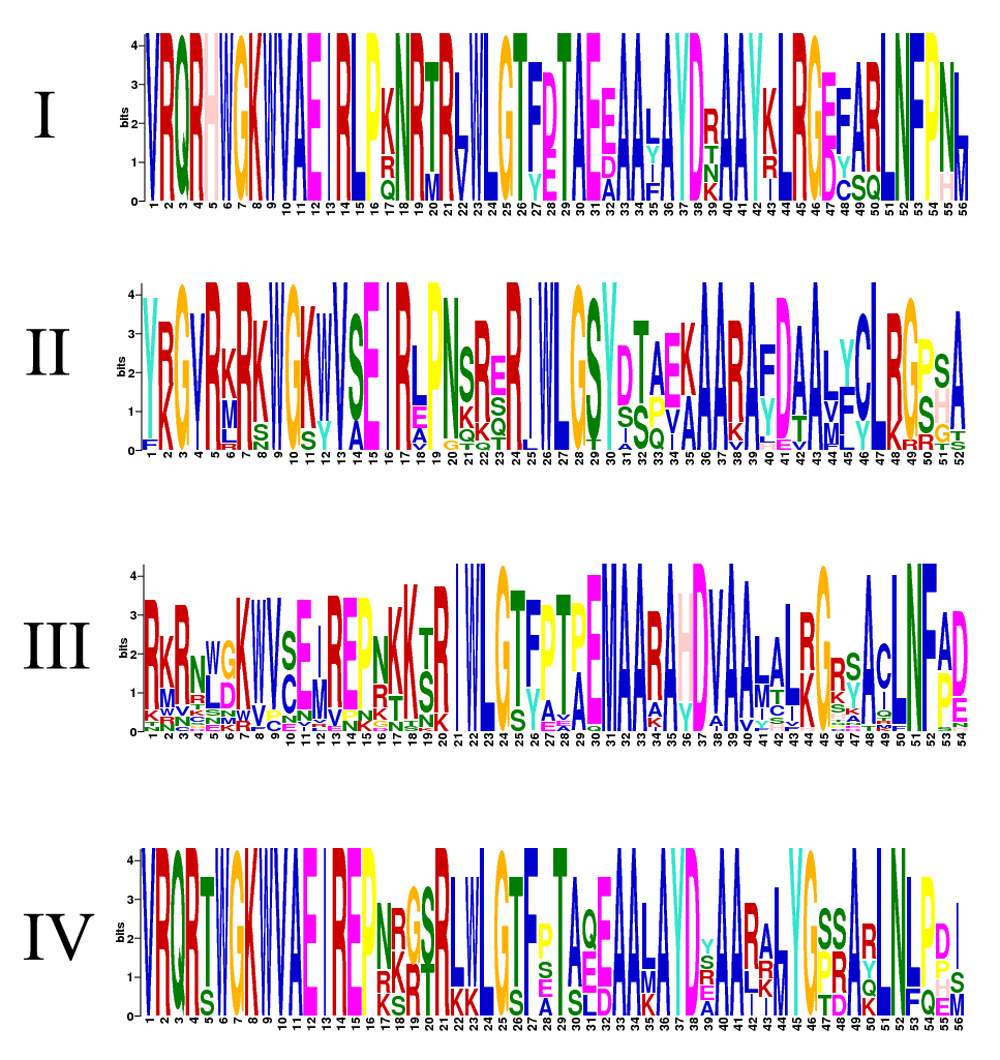

Supplement: Figure S1 — Conserved motifs identified from members of the DREB subfamily in Medicago truncatula. [file Image1.JPEG]

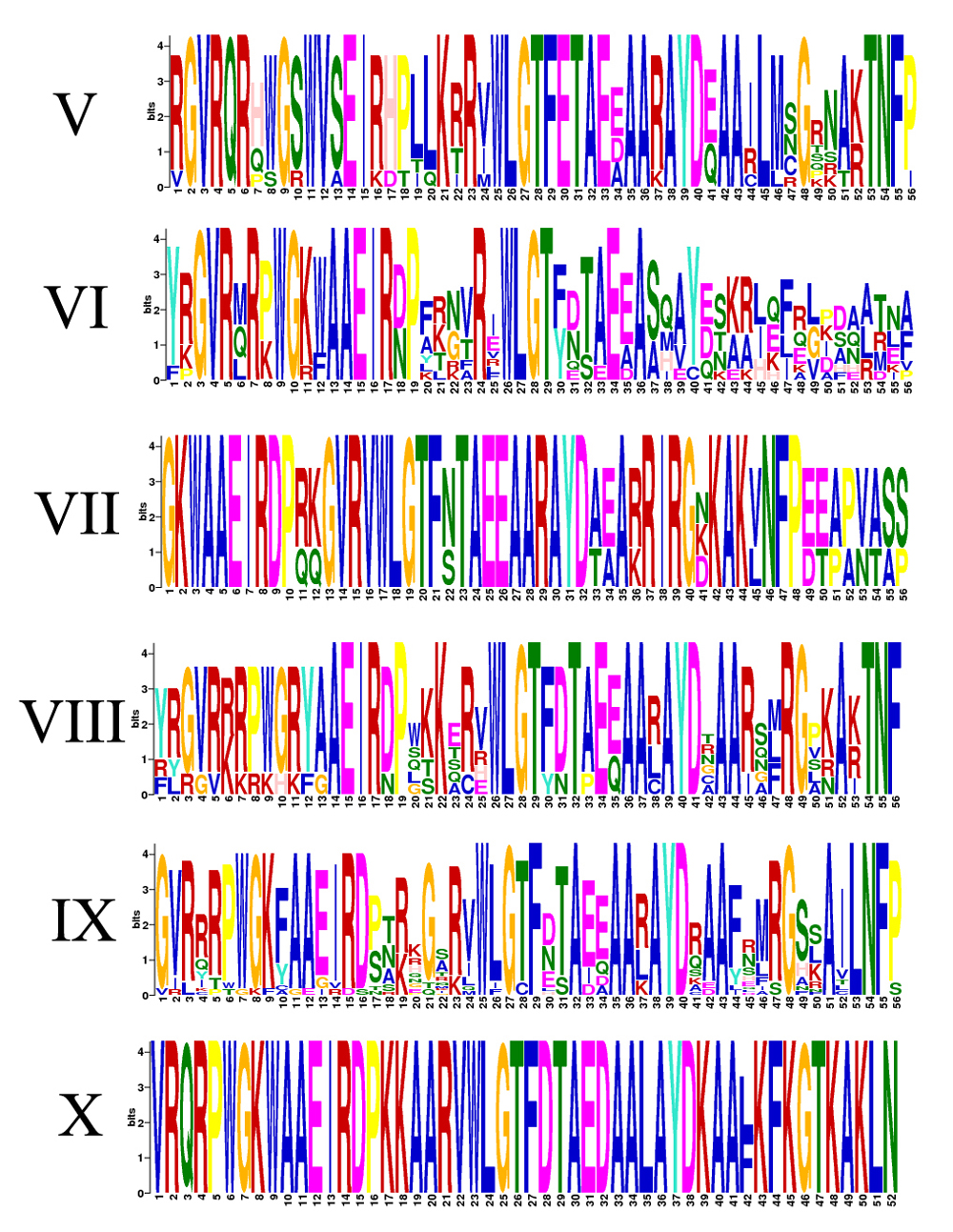

Supplement: Figure S2 — Conserved motifs identified from members of the ERF subfamily in Medicago truncatula. [file Image2.JPEG]

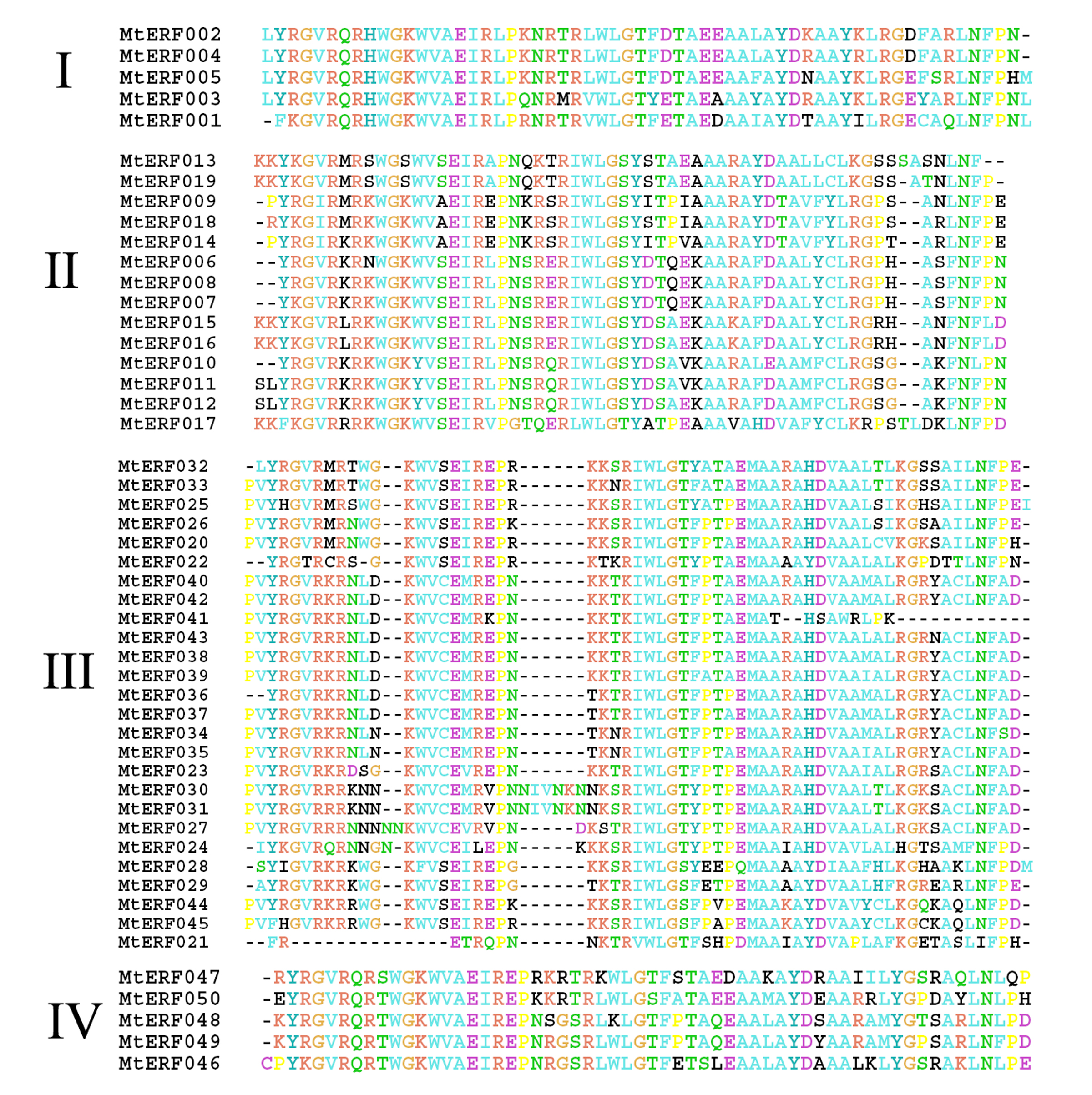

Supplement: Figure S3 — Comparison of amino acid sequences of the AP2/ERF domains in the DREB subfamily. [file Image3.JPEG]

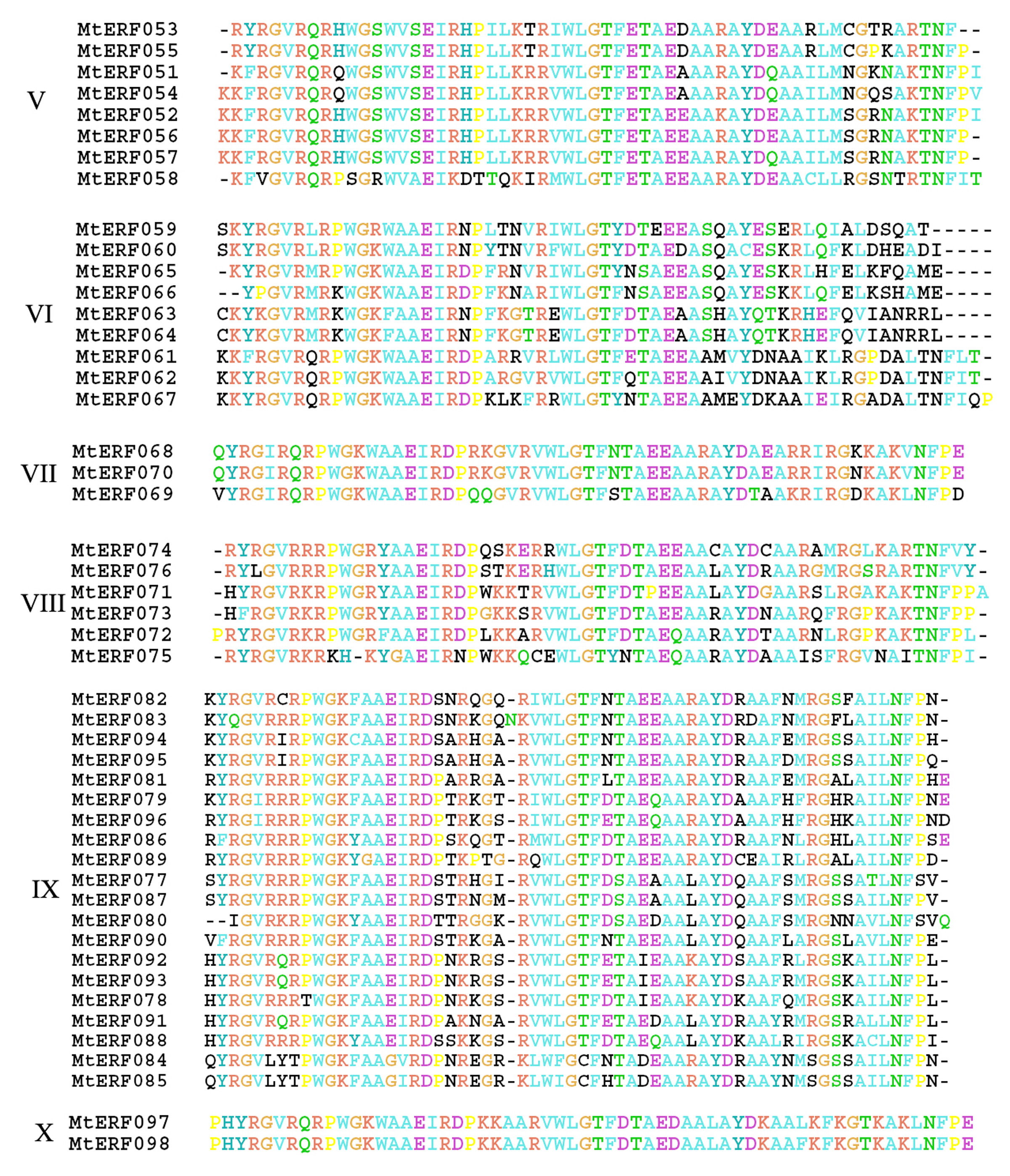

Supplement: Figure S4 — Comparison of amino acid sequences of the AP2/ERF domains in the ERF subfamily. [file Image4.JPEG]

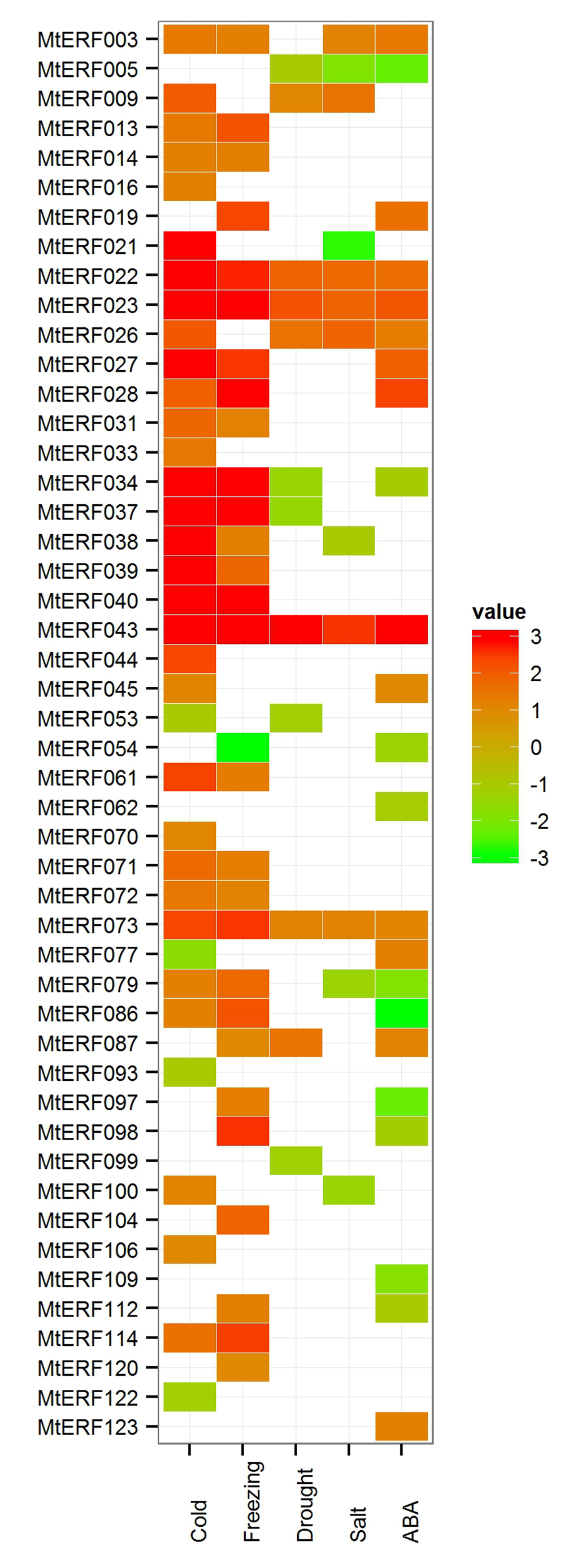

Supplement: Figure S5 — Differential expression analysis of MtERF genes involved in the response to abiotic stress. [file Image5.JPEG]

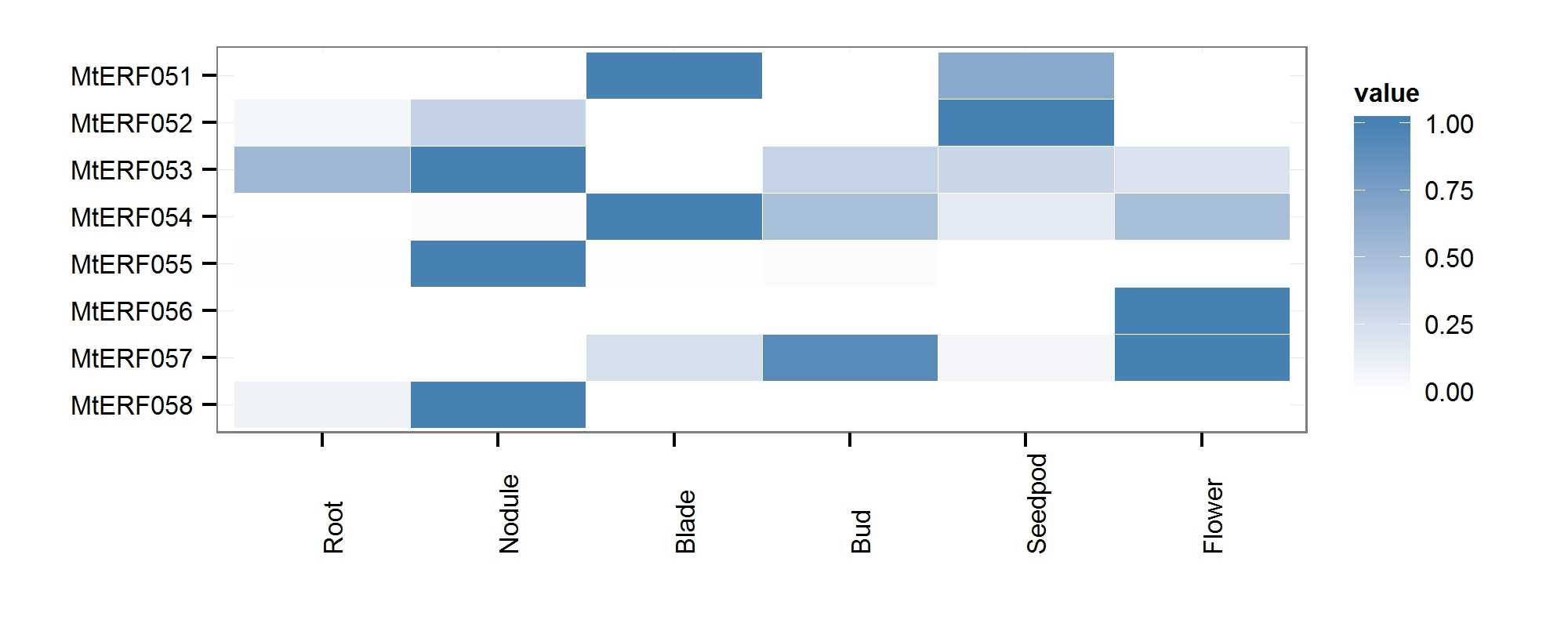

Supplement: Figure S6 — Expression profile cluster analysis members of MtERF V group in tissue development. [file Image6.JPEG]

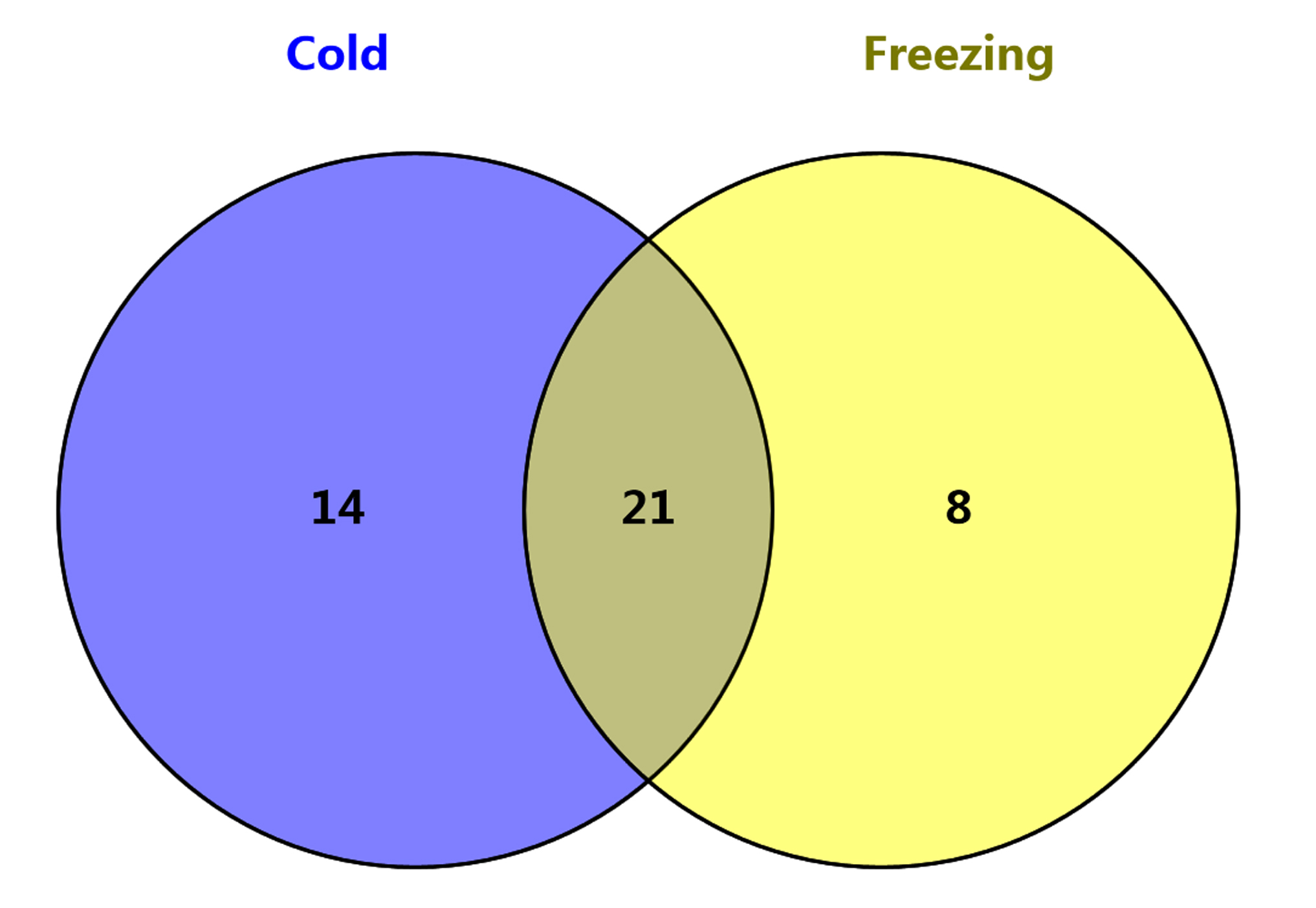

Supplement: Figure S7 — Venn diagram of shared expression MtERF genes between tissues development and stresses response. [file Image7.JPEG]

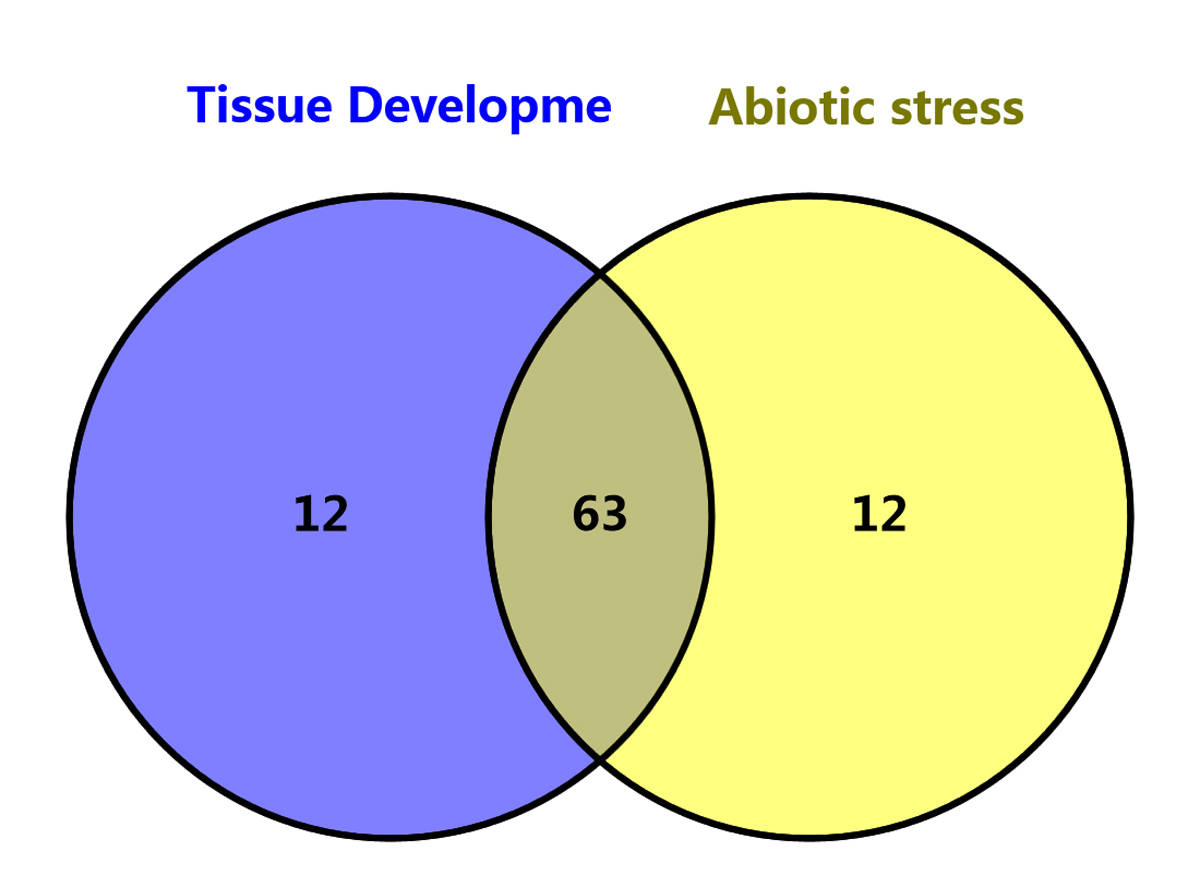

Supplement: Figure S8 — Venn diagram of shared expression MtERF genes between cold stress and freezing stress. [file Image8.JPEG]

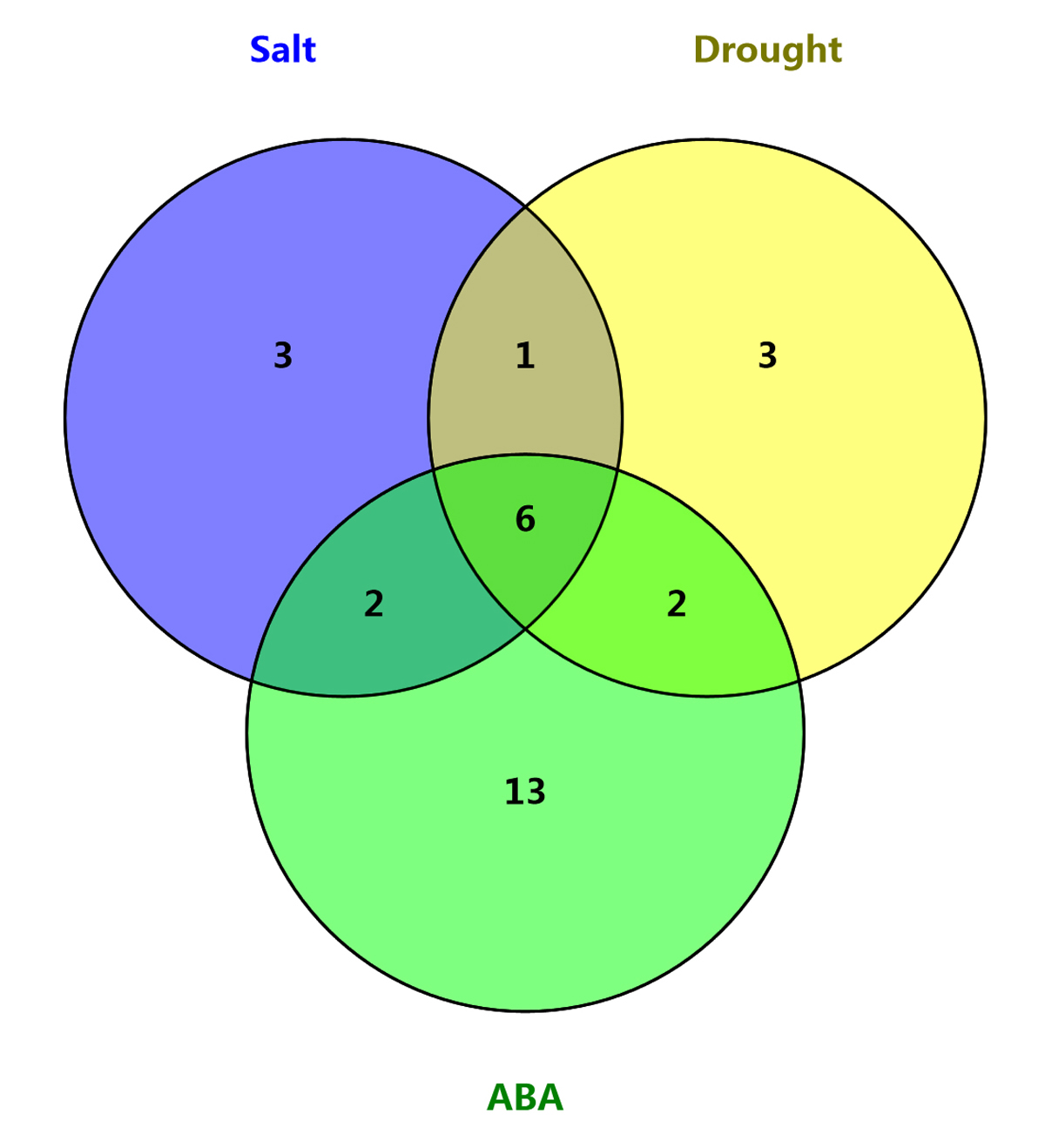

Supplement: Figure S9 — Venn diagram of shared expression MtERF genes among salt, drought and ABA stresses. [file Image9.JPEG]
