# Supplementary material for: Genome-Wide Analysis of the AP2/ERF Superfamily Genes and their Responses to Abiotic Stress in Medicago truncatula
Source: Front Plant Sci. 2016 Jan 19;6:1247. doi: 10.3389/fpls.2015.01247 (PMC4717309; doi:10.3389/fpls.2015.01247)
Supplement: Table S1 — List of qRT-PCR validation primers used in the present study. [file Table1.DOC]

Table S1 List of qRT-PCR validation primers used in the present study

| Gene | Forward primer | Reverses primer |
| --- | --- | --- |
| MtERF013 | TGGGTATCAGAGATTAGAGCA | TGCAGCTACTCTTTGAATTG |
| MtERF014 | GAAAGCAACAACAAACCTCA | AAATAGAATACGGCGGTGTC |
| MtERF021 | GCCTTTAGCTTTCAAAGGTG | ATAATCCGCAGACTCAGAAC |
| MtERF023 | TAAGAAAAAGGGATTCCGGG | GCCTTTTGAATATCCCTTGC |
| MtERF031 | AAGACCAAAGAAGAGAGCAG | AAGAGTAAGTGCAGCAACAT |
| MtERF038 | AGAAGTTTAAGGAGACTCGC | AGTCTGCAAAGTTGAGACAG |
| MtERF039 | TAGGATTTGGCTAGGGACTT | AGTCTTGTCTGGTCTGAAAG |
| MtERF043 | GGAGGAAGAAGTTCAAGGAG | AGGCAGAGTCAGCAAAATTA |
| MtERF044 | AAATCTCGCATATGGCTAGG | ACGAAGCCTTCATCATACTC |
| GAPDH | TAAGGGTGGTGCCAAGAAGGT | AGCAAGAGGAGCAAGGCAGTT |
