# Supplementary material for: Genome-Wide Analysis of the AP2/ERF Superfamily Genes and their Responses to Abiotic Stress in Medicago truncatula
Source: Front Plant Sci. 2016 Jan 19;6:1247. doi: 10.3389/fpls.2015.01247 (PMC4717309; doi:10.3389/fpls.2015.01247)
Supplement: Table S2 — Summary of miR172 targeting AP2/ERF TF genes in Medicago truncatula. [file Table2.DOC]

Table S2 Summary of miR172 targeting AP2/ERF TF genes in *Medicago truncatula*

| miRNA_Acc. | Target_Acc. | Expectation | UPE | miRNA_start | miRNA_end | Target_start | Target_end | miRNA_aligned_fragment | Target_aligned_fragment | Inhibition |
| --- | --- | --- | --- | --- | --- | --- | --- | --- | --- | --- |
| mtr-miR172a | MtERF101 | 0 | 13.881 | 1 | 21 | 1453 | 1473 | AGAAUCCUGAUGAUGCUGCAG | CUGCAGCAUCAUCAGGAUUCU | Cleavage |
| mtr-miR172a | MtERF108 | 1 | 12.913 | 1 | 21 | 1469 | 1489 | AGAAUCCUGAUGAUGCUGCAG | CUGCAGCAUCAUCAGGAUUCC | Cleavage |
| mtr-miR172a | MtERF113 | 1 | 17.208 | 1 | 21 | 1828 | 1848 | AGAAUCCUGAUGAUGCUGCAG | CUGCAGCAUCAUCAGGAUUCC | Cleavage |
| mtr-miR172a | MtERF106 | 1 | 12.833 | 1 | 21 | 1733 | 1753 | AGAAUCCUGAUGAUGCUGCAG | CAGCAGCAUCAUCAGGAUUCU | Cleavage |
| mtr-miR172b | MtERF101 | 0.5 | 13.881 | 1 | 20 | 1454 | 1473 | AGAAUCUUGAUGAUGCUGCA | UGCAGCAUCAUCAGGAUUCU | Cleavage |
| mtr-miR172b | MtERF106 | 1.5 | 12.833 | 1 | 20 | 1734 | 1753 | AGAAUCUUGAUGAUGCUGCA | AGCAGCAUCAUCAGGAUUCU | Cleavage |
| mtr-miR172b | MtERF113 | 1.5 | 17.208 | 1 | 20 | 1829 | 1848 | AGAAUCUUGAUGAUGCUGCA | UGCAGCAUCAUCAGGAUUCC | Cleavage |
| mtr-miR172b | MtERF108 | 1.5 | 12.913 | 1 | 20 | 1470 | 1489 | AGAAUCUUGAUGAUGCUGCA | UGCAGCAUCAUCAGGAUUCC | Cleavage |
| mtr-miR172c-3p | MtERF101 | 0.5 | 13.881 | 1 | 20 | 1454 | 1473 | AGAAUCUUGAUGAUGCUGCA | UGCAGCAUCAUCAGGAUUCU | Cleavage |
| mtr-miR172c-3p | MtERF106 | 1.5 | 12.833 | 1 | 20 | 1734 | 1753 | AGAAUCUUGAUGAUGCUGCA | AGCAGCAUCAUCAGGAUUCU | Cleavage |
| mtr-miR172c-3p | MtERF113 | 1.5 | 17.208 | 1 | 20 | 1829 | 1848 | AGAAUCUUGAUGAUGCUGCA | UGCAGCAUCAUCAGGAUUCC | Cleavage |
| mtr-miR172c-3p | MtERF108 | 1.5 | 12.913 | 1 | 20 | 1470 | 1489 | AGAAUCUUGAUGAUGCUGCA | UGCAGCAUCAUCAGGAUUCC | Cleavage |
| mtr-miR172d-3p | MtERF101 | 0.5 | 13.881 | 1 | 20 | 1454 | 1473 | AGAAUCUUGAUGAUGCUGCA | UGCAGCAUCAUCAGGAUUCU | Cleavage |
| mtr-miR172d-3p | MtERF108 | 1.5 | 12.913 | 1 | 20 | 1470 | 1489 | AGAAUCUUGAUGAUGCUGCA | UGCAGCAUCAUCAGGAUUCC | Cleavage |
| mtr-miR172d-3p | MtERF113 | 1.5 | 17.208 | 1 | 20 | 1829 | 1848 | AGAAUCUUGAUGAUGCUGCA | UGCAGCAUCAUCAGGAUUCC | Cleavage |
| mtr-miR172d-3p | MtERF106 | 1.5 | 12.833 | 1 | 20 | 1734 | 1753 | AGAAUCUUGAUGAUGCUGCA | AGCAGCAUCAUCAGGAUUCU | Cleavage |
